# Insights Into the Pathological Glycosylation Associated With COG6-CDG

**DOI:** 10.1155/humu/7948771

**Published:** 2025-11-30

**Authors:** Zuzana Pakanová, Maroš Krchňák, Marek Nemčovič, Rebeka Kodríková, Nina Ondrušková, Hana Štufková, Mária Giertlová, Katarína Okáľová, Paula Stretavská, Slavomíra Martineková, Renáta Zemjarová Mezenská, Michaela Urminská, Martina Škopková, Andrea Andrésová, Miroslava Lysinová, Lenka Belujská, Anna Šalingová, Gábor Beke, Lucia Račková, Tomáš Honzík, Hana Hansíková, Peter Baráth

**Affiliations:** ^1^Department of Glycobiology, Institute of Chemistry, Slovak Academy of Sciences, Bratislava, Slovakia; ^2^Department of Pediatrics and Inherited Metabolic Disorders, First Faculty of Medicine, Charles University and General University Hospital in Prague, Prague, Czech Republic; ^3^Department of Neurology, Faculty of Medicine, Pavol Jozef Šafárik University, Košice, Slovakia; ^4^Department of Clinical Neurosciences, Center of Clinical and Preclinical Research MEDIPARK, Pavol Jozef Šafárik University, Košice, Slovakia; ^5^Ambulance of Medical Genetics, Children's Faculty Hospital and Slovak Health University, Banská Bystrica, Slovakia; ^6^Ambulance of Medical Genetics, Unilabs Slovakia, Košice, Slovakia; ^7^Pediatric Neurology Ambulance, Children's Faculty Hospital With Clinic Banská Bystrica, Banská Bystrica, Slovakia; ^8^Ambulance of Medical Genetics, Unilabs Slovakia, Banská Bystrica, Slovakia; ^9^Department of Medical Genetics, F.D. Roosevelt University Hospital With Policlinic Banská Bystrica, Banská Bystrica, Slovakia; ^10^Institute of Biology and Medical Genetics, First Faculty of Medicine, Charles University and General University Hospital in Prague, Prague, Czech Republic; ^11^Laboratory of Medical Genetics, Unilabs Slovakia, Bratislava, Slovakia; ^12^Institute of Experimental Endocrinology, Biomedical Research Center, Slovak Academy of Sciences, Bratislava, Slovakia; ^13^The Ambulance of Pediatric Endocrinology and Diabetology and Metabolic and Nutritional Disorders, Children's Faculty Hospital With Clinic Banská Bystrica, Banská Bystrica, Slovakia; ^14^Department of Laboratory Medicine, National Institute of Children's Diseases, Bratislava, Slovakia; ^15^Institute of Molecular Biology, Slovak Academy of Sciences, Bratislava, Slovakia

**Keywords:** COG6-CDG, glycomics, glycoprofile, mass spectrometry

## Abstract

**Background and Aims:**

Congenital disorders of glycosylation (CDG) are rare diseases caused by defects in protein glycosylation. We present an infant with multisystemic clinical involvement, diagnosed with COG6-CDG.

**Methods:**

Serum and transferrin-linked N-glycans, as well as serum and apolipoprotein CIII–linked O-glycans, were analyzed by MALDI mass spectrometry. Mutation analysis was performed by next-generation sequencing. Functional studies assessed COG6 subunit expression, cooperating subunits, and retrograde transport. GlycoWorks RapiFluor-MS–based N-glycan labeling with HPLC-FLD and ESI-Orbitrap mass spectrometry enabled further comprehensive glycoprofile analysis.

**Results:**

Aberrant glycosylation typical of combined N- and O-glycosylation defects was detected. Mutation analysis identified a novel homozygous variant in the *COG6* gene: c.906_907delinsA, p.(His302GlnfsTer4), introducing a premature stop codon and producing a truncated protein of only 304 amino acids. The diagnosis of COG6-CDG was confirmed by the complete absence of the COG6 subunit, impairment of two other cooperating subunits, and delayed retrograde transport. Independent glycoprofile analyses by HPLC-FLD and ESI-Orbitrap revealed a set of potential glycobiomarkers of COG6-CDG, including underprocessed N-glycans Hex3-5HexNAc2, Hex3-5HexNAc3, Hex3-4HexNAc4, and Hex4HexNAc3-4NeuAc1.

**Conclusion:**

This study describes a novel COG6 variant leading to complete loss of protein function and major glycosylation abnormalities. Multiomics analysis provided deeper insights into the molecular mechanisms of this rare disease and the function of the *COG6* gene and demonstrated how the mutation results in significant alterations in the patient's (glyco)phenotype.

## 1. Introduction

The conserved oligomeric Golgi (COG) complex is involved in intracellular transport and glycoprotein modifications in the Golgi apparatus. It is a hetero-octamer containing eight different subunits that are organized into two lobes—Lobe A, consisting of COG1–4, and Lobe B, consisting of COG5–8 [1, 2]—and it contributes to membrane trafficking of proteins within the Golgi and to retrograde transport from the Golgi to the endoplasmic reticulum (ER). If the balance between anterograde and retrograde transport of the resident Golgi proteins is disturbed, the glycosylation machinery is mislocalized and can cause glycosylation defects [3]. Dysfunction of the COG complex leads to the separation of glycosyltransferases from anterograde cargo molecules passing along the secretory pathway, thus affecting normal protein glycosylation [4].

Defects in COG subunits manifest as a class of human diseases known as congenital disorders of glycosylation (CDG) Type II [5]. The first COG6-CDG patient was described in 2010 [6], and based on our knowledge, 33 patients with COG6-CDG have been reported up to this date [2, 6–21]. Both the number of patients and the genetic and phenotypic spectrum of this glycosylation disorder are continuously growing. To date, no publication has comprehensively focused on the precise determination of the glycoprofile of COG6-CDG using a combination of analytical methods, including HPLC as a commonly used quantitative method with MALDI and ESI mass spectrometry (MS), enabling precise structural analysis and identification of potential biomarkers.

COG-CDG clinical presentation includes developmental disability, hypotonia, microcephaly, dysmorphic features, skin abnormalities, skeletal anomalies, liver and gastrointestinal dysfunction, hematological abnormalities, and congenital malformations [10]. The genotype–phenotype relationship was demonstrated in several studies, ranging from homozygous nonsense and frameshift mutations resulting in early lethality [7] to compound heterozygous variants and deep intronic splice site mutations as the mildest COG6-CDG form in the subgroup of patients known as Shaheen syndrome [2, 17]. To enable a deeper understanding of the pathological molecular mechanisms of this disease, the description of each novel variant, including its impact on the clinical and biochemical phenotype and a comprehensive description of the glycoprofile, is crucial. Furthermore, a multiomics approach can lead to an effective characterization of underlying processes that are ongoing in individual CDG subtypes.

## 2. Methods

### 2.1. Samples

Serum samples were obtained from the National Institute of Children's Diseases (Bratislava, Slovakia) under standard operating procedures. As a control, commercially available human serum (Merck, DE) or serum from healthy volunteers was used. The study was approved by the hospital's Ethics Committee under the Registration Number EK1/3/2023. For MS-based experiments, both the patient's serum sample and control were analyzed in two replicates.

The patient's fibroblast cell line was established from a cryopreserved umbilical cord tissue stored in a cord blood bank. The use of umbilical cord material, collected at birth, was primarily due to the limited availability of patient-derived biological samples, as the patient had already exited at the time of our investigations. The umbilical cord tissue cut into small pieces was cultured in a reduced serum (2%) MesenPro RS Medium containing antibiotics during the initial outgrowth of cells and the first three passages. Further passages were transferred to standard 10% FBS-containing DMEM with antibiotics at 37°C in a 5% CO_2_ environment. Control dermal fibroblasts from two male newborns as the age-matched controls were purchased commercially from ATCC (VA, United States) (named as “Control 1”) and one control obtained from a healthy female adult volunteer (named as “Control 2”).

### 2.2. Mutation Analysis and In Silico Analysis

#### 2.2.1. Next-Generation Sequencing

Genomic DNA extraction and sequencing library preparation were performed following the manufacturers' instructions. Using the NGS method with a hybridization solution (Illumina DNA Prep with Enrichment, TruSight One Expanded; Illumina, CA, United States), the examined individual's clinical exome sequencing (CES) was specifically enriched and sequenced. The CES was targeted to specific regions of interest (ROIs) as defined by the manufacturer. The analysis focused on selected genes, their coding, and adjacent splicing-relevant regions (minimum −5/+5). Sequencing libraries that passed quality control were sequenced on Illumina NextSeq 550 (Illumina, CA, United States) using paired-end sequencing (2 × 100 bp). The obtained sequencing data were analyzed using Illumina bcl2fastq v2.20 and Emedgene Analyze software (Illumina, CA, United States), referencing the *Homo sapiens* genome (GRCh38) and the RefSeq database. The variants were entered using the HGVS nomenclature [22] with their classification based on the available ACMG information and recommendations [23, 24].

To investigate the impact of a deletion–insertion mutation on the amino acid sequence, we retrieved the coding sequence of the target *COG6* gene from RefSeq (Accession No. NM_020751.3). To demonstrate the impact of the mutation (c.906_907delinsA, p.(His302GlnfsTer4)) on the amino acid sequence length, the ExPASy Translate tool was used [25, 26].

#### 2.2.2. Sanger Sequencing of the Target Region of the *COG6* Gene

The target region of the *COG6* gene (exon 9; NM_020751.3) was amplified by forward primer 5⁣′-GCGTGAATAGTCTGATATAGCC-3⁣′ and reverse primer 5⁣′-GAACAGAAACAGAGATGCTTGT-3⁣′ using polymerase chain reaction (PCR). Sanger sequencing of the PCR product was performed on an ABI PRISM 3130XL Analyzer (Applied Biosystems, MA, United States/Hitachi, Japan). The obtained data were analyzed using the Geneious Prime software program (Biomatters Ltd., New Zealand).

#### 2.2.3. *COG6* mRNA Sequencing

RNA was extracted from patient and control cultured fibroblasts using the RNeasy Mini Kit (Qiagen, Germany) and reverse-transcribed using SuperScript III Reverse Transcriptase (Invitrogen, MA, United States). The region of exons 7–12 of the NM_020751.3 *COG6* cDNA was amplified using 5⁣′-ACAAATCAACAAACGGCAGGT-3⁣′ and 5⁣′-GCCCCAGGTTCAGCAACTAT-3⁣′ forward and reverse primers, respectively. Sequencing was performed using the ABI PRISM 3500 Analyzer (Applied Biosystems, MA, United States/Hitachi, Japan), and data were analyzed using SeqScape v2.7 (Applied Biosystems, MA, United States).

### 2.3. Electrophoresis and Western Blot of COG Subunits and LAMP2 Protein

To prepare the cell lysates, the fibroblasts were rinsed twice with room temperature PBS, harvested in ice-cold PBS by scraping, and processed as described previously [27]. SDS-PAGE was carried out under standard conditions with 12% polyacrylamide or gradient 6%–15% gel (for LAMP2 and COG6 analysis) and 0.1% SDS gels and transferred onto Immobilon-P PVDF Membrane (Millipore, MA, United States) by semidry electroblotting using the Hoefer Semi-Dry Transfer Unit (Harvard Bioscience, MA, United States). The following primary antibodies were used to detect the proteins of interest: mouse monoclonal antibodies against COG1 (Abcam, United Kingdom, 1:2000), COG4 (Abcam, United Kingdom, 1: 2000), COG6 (aa558-658, Abcam, United Kingdom, 1:5000), COG7 (Abcam, United Kingdom, 1:2000), LAMP2 (Santa Cruz Biotechnology, TX, United States, 1:20,000), and *β*-Actin (Cell Signaling, MA, United States, diluted 1:10,000). Blots were incubated with primary antibodies in TBS, 0.1% Tween 20, and 1% nonfat dried milk for 2 h. As the secondary antibodies, peroxidase-conjugated Anti-Mouse IgG (whole molecule) produced in goat (Sigma-Aldrich, MA, United States, dilution 1:2000) or peroxidase-conjugated Anti-Rabbit IgG (whole molecule) produced in goat (Sigma-Aldrich, MA, United States, dilution 1:2000) was used. Membranes were incubated with secondary antibodies in TBS, 0.1% Tween 20, and 1% nonfat dried milk for 1 h. The blots were developed with SuperSignal West Femto Maximum Sensitivity Substrate (Thermo Fisher Scientific, MA, United States) using Syngene G:Box (Syngene, India).

### 2.4. Analysis of the Golgi Structure and Trafficking by Fluorescence Microscopy

The fibroblasts were cultured to the confluency of approximately 70%. For Golgi structure analysis, the cells were rinsed and fixed with 4% paraformaldehyde, and the cell membranes were permeabilized with 0.1% Triton X-100 in PBS. After blocking with 5% FBS in PBS, the samples were incubated overnight at 4°C with a mouse monoclonal Anti-Giantin Antibody (Abcam, United Kingdom, 1:100 dilution in blocking solution). The next day, the fluorescent-labeled secondary antibody (anti-Mouse IgG1-Alexa Fluor 488, Invitrogen, MA, United States, diluted 1:500 in the blocking solution) was added for 2 h at room temperature, followed by nucleus staining with DAPI solution (Invitrogen, MA, United States, 10 *μ*g/mL in PBS). For the brefeldin A (BFA) assay, the culture medium for the cells on coverslips was replaced by medium containing BFA (Sigma-Aldrich, MA, United States, 2.5 *μ*g/mL), and the reaction was stopped after 12 min by washing the fibroblasts with ice-cold PBS. Finally, the signals from blue and green channels (excitation filters 377/50 and 466/40 and emission filters 447/60 and 520/50 nm, respectively) were recorded using the ECLIPSE Ti2-U epifluorescence microscope (Nikon, Japan) with NIS-Elements imaging software (Nikon, Japan, Version 5.11) at manually set exposure time. Multiple images were acquired for each experiment to evaluate the giantin signal distribution. The percentage of cells with Golgi remnants was calculated in > 100 cells from different fields.

### 2.5. Analysis of Transferrin (Tf) Glycosylation Pattern

Analysis of Tf glycosylation pattern by isoelectric focusing (IEF) was performed using PhastSystem (GE HealthCare, Chicago, IL) as described in our previous work [28]. Tf was further isolated from 50 *μ*L of serum using an immunoaffinity chromatography column prepared by binding rabbit polyclonal anti-Tf antibodies (Dako, Germany) on CNBr-Sepharose according to the manufacturer's instructions. After washing with PBS, Tf was eluted by 0.2-M glycine, pH 2.7, and the eluates were immediately neutralized by adding 1-M Tris, pH 8.5. The quality and purity of isolated Tf were determined by MALDI-TOF analysis of the eluate in linear positive ion mode using Ultraflextreme II mass spectrometer (Bruker, MA, United States) after its desalting by ZipTips (Millipore, MA, United States) according to the manufacturer's instructions. Analysis of Tf N-glycoprofile was performed as described below (Section 2.6).

### 2.6. Analysis of Serum N-Glycoprofile by MALDI-TOF MS

Analysis of serum N-glycoprofile by MALDI-TOF MS was performed as described previously [29]. Relative intensities were calculated for each individual glycan structure with normalization to the sum of all the assigned peaks.

### 2.7. Analysis of Serum O-Glycoprofile and Apolipoprotein CIII (ApoCIII) O-Glycoprofile by MALDI-TOF MS

Analysis of the O-glycoprofile of ApoCIII was performed as described in our previous work [30]. Relative intensities were calculated for each ApoCIII glycoisoform with normalization to the sum of all the assigned peaks.

To analyze serum O-glycoprofile, proteins were de-N-glycosylated [29] and precipitated using Wessel–Flüegge's extraction. The purified proteins, enriched with 5 *μ*L of 1250 pmol of internal standard (raffinose), were then subjected to *β*-elimination for the release of O-glycans, using 1-M KBH_4_ solution in 0.1-M KOH. The reaction was incubated overnight at 45°C and terminated by adding glacial acetic acid. Released O-glycans were purified by cation exchange (LudgerClean CEX cartridges, Ludger, United Kingdom), followed by purification through Supelclean ENVI-Carb SPE Tubes (Supelco, PA, United States) and permethylation. Samples were analyzed in reflectron positive ion mode using Ultraflextreme II mass spectrometer (Bruker, MA, United States) with manual interpretation. Relative intensities normalized to the intensity of internal standard were calculated for each O-glycan isoform.

### 2.8. Analysis of Serum N-Glycoprofile by HPLC-FLD Connected to ESI-Orbitrap MS

N-glycans from 1 *μ*L of serum were released and labeled with RapiFluor-MS reagent using a GlycoWorks RapiFluor-MS N-Glycan Kit (Waters, MA, United States) according to the manufacturer's instructions. Labeled N-glycans were separated on Dionex UltiMate 3000 UHPLC System (Thermo Scientific, MA, United States) with ACQUITY UPLC BEH Amide Column, 130 Å (1.7 *μ*m, 2.1 × 150 mm; Waters, MA, United States), utilizing gradient elution of 100% acetonitrile (MP A) and 50-mM ammonium formate (pH 4.4, MP B) at 60°C. The starting conditions were 25% MP B at 0.3 mL/min, which increased to 33% MP B in 2 min and 44% MP B in 35 min. Half the flow was directed to the Dionex 3100 Fluorescence Detector set to 265/425 nm (excitation/emission wavelengths). The second half was directed to the Orbitrap Elite mass spectrometer (Thermo Scientific, MA, United States) with a heated ESI probe (capillary temperature 350°C, source temperature 300°C, and source voltage 5 kV). Full scan spectra were acquired in positive ion mode in the *m/z* range of 500–2000. The top three most intense precursor ions were selected for collision-induced dissociation (CID) with a normalized collision energy of 35%. The obtained spectra were analyzed and deconvoluted (*z* = 1–3) using the Freestyle software program (v. 1.8.63.0, Thermo Scientific, MA, United States) with manual annotation (Δ*m*/*z* < 0.01 from theoretical values). Relative intensities were calculated for each individual glycan structure with normalization to the sum of all the assigned peaks.

## 3. Results

### 3.1. Clinical Presentation

The child, a female with an inapparent family history, was born prematurely at 36 weeks' high-risk gestation. The newborn was hypotrophic and dependent on respiratory support. The sucking reflex and reaction to sound and visual stimuli were absent. Neurological examination documented hypotonia with absent reflexes, oculomotor disturbance, anisocoria, and severe developmental delay. In addition, several congenital anomalies were observed: abnormal posture of the upper limbs with arachnodactyly, progressive microcephaly with brachycephaly and flat back of the head, high forehead, hypertelorism and telecanthus, antimongoloid palpebral fissure, hypoplastic nose, long philtrum, small mouth with narrow lips, dysplastic posteriorly rotated ears, and short neck with skinfold. Fixed contractures were present on both hands, with no skin creases and a hypoplastic thumb. Brain ultrasound findings included enlarged lateral ventricles, cysts of the choroid plexus, and progressive widening of the interhemispheric fissure and intraparenchymal cyst. Echocardiography detected open foramen ovale. Several episodes of unexplained hyperthermia without laboratory markers of inflammation accompanied by agitation and severe anemia with anisocytosis evolved with the need for repeated erythrocyte transfusions, while mild thrombocytopenia recovered spontaneously. Several infectious complications occurred, including pneumonia.

In biochemical findings, hepatopathy was observed with elevated ALT, AST, and GGT. Furthermore, hyperlactatemia and hyperammonemia were detected with extremely elevated alpha-fetoprotein, decreased vitamin D, laboratory signs of coagulopathy, and increased ALP levels. Coagulation abnormalities mainly included decreased Factor XI and antithrombin, as well as an elevated APTT ratio. At the age of 4 weeks, significant transient elevations of troponin and creatine kinase were observed, without signs of cardiomyopathy, myopathy, or, eventually, neuropathy. The patient died of cardiopulmonary insufficiency at the age of 15 weeks.

### 3.2. Mutation Analysis and In Silico Analysis

**Figure 1:**
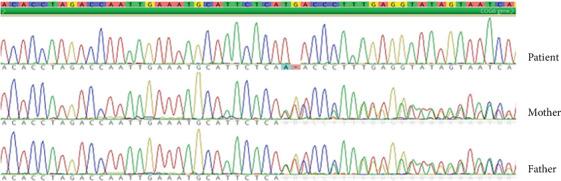
The likely pathogenic variant c.906_907delinsA, p.(His302GlnfsTer4) in exon 9 in the *COG6* gene in homozygous state in the patient and heterozygous state in the patient's mother and father verified by Sanger sequencing. Deletion of thymine and guanine at positions 906 and 907, followed by adenine insertion, results in a premature stop codon.

A targeted, comprehensive panel for arthrogryposis and skeletal dysplasias revealed a homozygous variant c.906_907delinsA, p.(His302GlnfsTer4) in exon 9 in the *COG6* gene (NM_020751.3) located on chromosome 13q14.11, inherited from both the patient's father and mother. The deletion of thymine and guanine at positions 906 and 907, followed by the insertion of adenine, leads to the formation of a premature TGA stop codon (Figure 1). This variant has not been previously described in the literature and is not included in the population or pathogenic variant databases. *COG6* mRNA sequencing (data not shown) excluded the usage of alternative splice sites and confirmed the presence of the transcript with a premature stop codon in exon 9 of 19, which should lead to its degradation in the nonsense-mediated decay pathway. Even if expressed, a severely truncated protein would be produced, leading to a loss of normal function. After the translation of mutated DNA into the amino acid sequence, changes in amino acids located at positions 302–304, together with a frameshift leading to a stop codon, were identified. As a result, a truncated protein consisting of only 304 amino acids is created (Figure S1) instead of the original protein composed of 657 amino acids (UniProt ID: Q9Y2V7).

According to the standards and guidelines recommended by the ACMG [27], this mutation is likely pathogenic.

### 3.3. SDS-PAGE/Western Blot of COG Subunits and LAMP2

**Figure 2:**
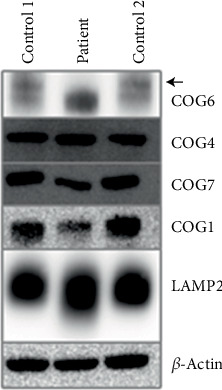
SDS-PAGE/Western blot images of COG1, COG4, COG6 (arrow), and COG7 proteins in the fibroblasts of a COG6-CDG patient, commercially available control (Control 1) and fibroblasts from an adult healthy individual (Control 2). Proteins were resolved by SDS-PAGE, electroblotted, and probed with specific antibodies. *β*-actin, internal loading control; LAMP2, late endosomal/lysosomal marker.

SDS-PAGE/Western blot analysis of cultured patient fibroblasts revealed an absence of COG6 protein compared with controls (Figure 2). The anti-COG6 antibody was raised against a recombinant fragment corresponding to amino acids 558–658; therefore, the truncated protein present in the patient sample could not be detected (the lower band observed represents a nonspecific antibody artifact). Densitometric analysis confirmed reduced levels of COG7 and COG1 proteins, by approximately 80% and 40%, respectively, whereas COG4 protein levels were not significantly altered. In contrast, the patient showed a markedly increased LAMP2 antibody signal (190%) with a slight shift in migration.

### 3.4. Analysis of the Golgi Structure and Trafficking by Immunofluorescence Microscopy

**Figure 3:**
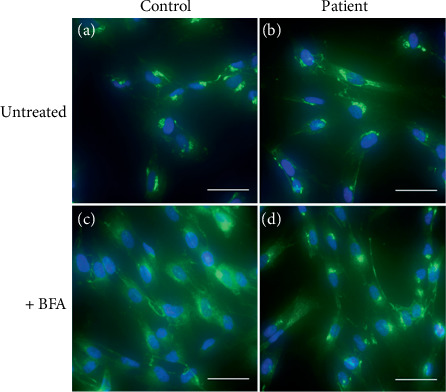
Analysis of the Golgi structure and trafficking by immunofluorescence microscopy in COG6-CDG patient and newborn control fibroblasts. To visualize the Golgi apparatus and analyze the Golgi retrograde transport, the *cis*-Golgi marker giantin was detected using immunocytochemistry and is shown in the green channel. Cell nuclei were stained with DAPI, which fluoresces in the blue channel. Analyses were performed in either (a, b) untreated or (c, d) BFA-treated fibroblasts (+ BFA, 2.5 *μ*g/mL, 12 min) from both the control and the patient. A delay in retrograde transport from the Golgi apparatus to the ER was confirmed in patient fibroblasts. Scale bar: 50 *μ*m.

Immunofluorescence microscopy was used to visualize the Golgi apparatus and to assess Golgi-to-ER retrograde trafficking induced by BFA treatment (Figure 3). No marked differences were observed in the Golgi structure between the untreated control and patient fibroblasts. The percentage of cells with Golgi remnants, including Golgi apparatus in the early stage of tubule formation and in the later stage of overt tubulation with loss of ribbon structure [31], was determined in > 100 cells from different fields of vision in both lines treated with BFA (2.5 *μ*g/mL, 12 min); the corresponding values were 32% versus 96% in control versus patient fibroblasts, respectively, confirming that the patient cells exhibited a delay in the retrograde transport from the Golgi apparatus to the ER.

### 3.5. Analysis of Tf N-Glycosylation

**Figure 4:**
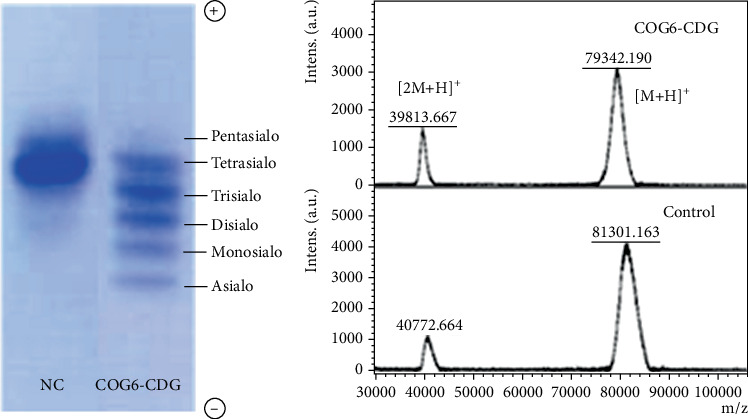
Glycosylation pattern of transferrin determined by (a) isoelectric focusing and (b) MALDI-TOF analysis of intact transferrin isolated from serum. A pathological pattern (Type II/II) was observed in the sample of the COG6-CDG patient. The molecular weight of intact transferrin isolated from the COG6-CDG patient was reduced by almost 2 kDa when compared with the control.

Analysis of the Tf glycosylation by IEF with immunofixation revealed a pathological Type II/II pattern (Figure 4a). Densitometric evaluation of individual fractions of the patient showed increased levels of asialo (9.9%), monosialo (15.9%), disialo (23.0%), and trisialo (26.1%), along with decreased levels of tetrasialo (21.0%) and pentasialo (4.9%) and with a completely absent hexiasialo-Tf in comparison with the control, suggesting a defect of N-glycan processing in the Golgi apparatus (CDG Type II). Analysis of the intact mass of the isolated patient's Tf revealed its decreased molecular weight when compared with the control (Figure 4b). The 2-kDa decrease in intact Tf mass could not be conclusively attributed to a specific structural alteration; therefore, complementary glycomic analyses were undertaken to elucidate the molecular basis of this mass shift.

Analysis of the patient's Tf N-glycans by MALDI-TOF MS confirmed the presence of aberrant underdeveloped structures (with corresponding relative intensities [%]: Hex3HexNAc3, 13.4%; Hex3HexNAc4Fuc0-1, 11.6% and 1.2%; Hex4HexNAc3Fuc0-1NeuAc1, 4.8% and 0.8%; Hex4HexNAc4, 9.7%; and Hex4HexNAc4NeuAc1, 4.8%). Furthermore, Hex5HexNAc2 N-glycan was identified at almost 20% of relative intensity, followed by trace amounts of Hex7HexNAc2 and Hex8HexNAc2 in the patient's Tf. These structures were not detected in control samples. An increased level of monosialylated biantennary Hex5HexNAc4NeuAc1 and decreased levels of disialylated biantennary Hex5HexNAc4NeuAc2 N-glycan were detected in the Tf of the COG6-CDG patient (Figure S2).

### 3.6. Analysis of Serum N-Glycoprofile

MALDI-TOF analysis of serum N-glycans revealed abnormal relative intensities of most of the 60 identified structures in the COG6-CDG patient. The patient's serum level of Hex3HexNAc3 was increased by almost 22-fold (Figure S3), whereas the level of Hex3HexNAc4 increased by nearly 16-fold. Additionally, the levels of oligomannose N-glycans (Hex3-Hex9HexNAc2) were increased compared with the control. Hex3HexNAc2 and Hex4HexNAc2 were not detected in controls, whereas their levels in the patient's serum reached 0.2% of relative intensity. The relative intensity of Hex5HexNAc2 increased more than sixfold, whereas the accumulation of another oligomannose glycans varied from almost twofold to fourfold. Furthermore, significantly increased relative levels of all hybrid N-glycans were detected, ranging from ninefold in the case of Hex4HexNAc3NeuAc1 to threefold in the case of Hex6HexNAc3NeuAc1. A significant decrease in the relative levels of disialylated biantennary Hex5HexNAc4NeuAc2 N-glycan (the most abundant glycan in healthy sera) and trisialylated triantennary Hex6HexNAc5Fuc0-2NeuAc3 N-glycan was detected in the COG6-CDG patient. Comprehensive data obtained from MALDI-TOF analysis of serum N-glycoprofile are shown in Table S1, and the representative mass spectra are shown in Figure S4. Increased levels of Hex5-7HexNAc2 oligomannose and underprocessed fucosylated Hex3HexNAc2-5Fuc1 and Hex4HexNAc3-4Fuc1 N-glycans altogether with decreased representation of disialylated structures were confirmed by MALDI-TOF analysis of the N-glycoprofile of the lysed fibroblasts. Furthermore, the sum of oligomannose N-glycans accounted for 55% in COG6-CDG fibroblasts and 44% in controls. Overall sialylation accounted for 14% in COG6-CDG and 29% in control. Data obtained from MALDI-TOF analysis of N-glycans isolated from lysed fibroblasts are shown in Table S2, and the representative mass spectra are shown in Figure S5.

**Figure 5:**
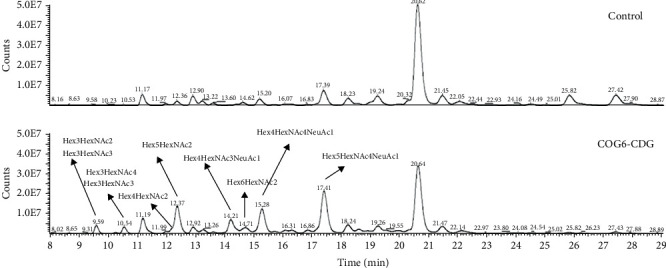
HPLC-FLD chromatogram of N-glycans isolated from the serum sample of COG6-CDG patient and control using the GlycoWorks RapiFluor-MS Kit. Arrows depict structures with increased levels in the COG6-CDG serum. Fuc, fucose; Hex, hexose; HexNAc, *N*-acetylglucosamine; NeuAc, sialic acid.

In addition to the permethylation and MALDI-TOF MS, serum N-glycans were analyzed by the GlycoWorks RapiFluor-MS approach, enabling both precise quantification and improved ionization in the positive ion mode of the ESI MS. Based on an extracted ion chromatogram (EIC), two isoforms of Hex3HexNAc3 (the first, major one eluting at 9.5 min and the second, minor one eluting at 10.5 min) were detected in the serum of the patient. However, the exact position of the single *N*-acetylglucosamine unit in these isoforms was not determined. This structure was not detected in the healthy control. Furthermore, the HPLC-FLD chromatogram confirmed increased relative levels of Hex3HexNAc4, oligomannose N-glycans, and hybrid Hex4HexNAc3NeuAc1 structure in the COG6-CDG sample. Additionally, the level of monosialylated biantennary Hex5HexNAc4NeuAc1 N-glycan was increased in the COG6-CDG sample, whereas the levels of disialylated biantennary Hex5HexNAc4NeuAc2 and trisialylated triantennary Hex6HexNAc5Fuc1NeuAc3 were decreased (Figure 5).

Due to the overlapping of fluorescence signals of individual glycans in HPLC-FLD, serum N-glycans labeled by the RapiFluor-MS tag were relatively quantified by ESI-Orbitrap MS. EICs were generated for 46 structures with unique molecular weights. Representative EICs of Hex3HexNAc3 and Hex3HexNAc4 are shown in Figure S6.

Based on ESI-Orbitrap analysis, the levels of Hex3-6HexNAc3 in the patient's serum accounted for 4.4%, 0.5%, 0.2%, and 0.2% of relative intensity, respectively, whereas these structures were not detected in the control (Table S3). Furthermore, the levels of Hex4HexNAc3NeuAc1 were increased more than ninefold, and Hex4HexNAc3Fuc1NeuAc1 was not detected in the control, whereas in the COG6-CDG patient's sample, it accounted for 0.3%. Both Hex5HexNAc3NeuAc1 and Hex6HexNAc3NeuAc1 levels were increased more than threefold and fourfold in COG6-CDG, respectively. Increased levels of almost all oligomannose N-glycans, including Hex3HexNAc2 (not identified in control) and Hex4HexNAc2, were also confirmed by the ESI MS–based approach. Hex3HexNAc3 N-glycan, considered one of the potential COG6-CDG glycobiomarkers in this study, was not detected in the control, and in the patient's serum, it accounted for 4.4% of relative intensity. The level of Hex3HexNAc4 was increased 13-fold, Hex4HexNAc4 more than fivefold, and Hex5HexNAc4 almost twofold in COG6-CDG, while their monosialylated forms Hex4-5HexNAc4NeuAc1 were also increased. Furthermore, the relative distribution of nearly all of the identified disialylated, trisialylated, and tetrasialylated N-glycans was evidently decreased in the COG6-CDG serum. The levels of the most abundant structure in the sera of controls, disialylated biantennary Hex5HexNAc4NeuAc2 N-glycan (relative intensity 55.2%), were decreased to 30.1% in the COG6-CDG patient. The datasets are deposited in the MS data repository for glycomics (GlycoPOST) under Project Identification No. GPST000459 [32].

### 3.7. Analysis of O-Glycoprofile

**Figure 6:**
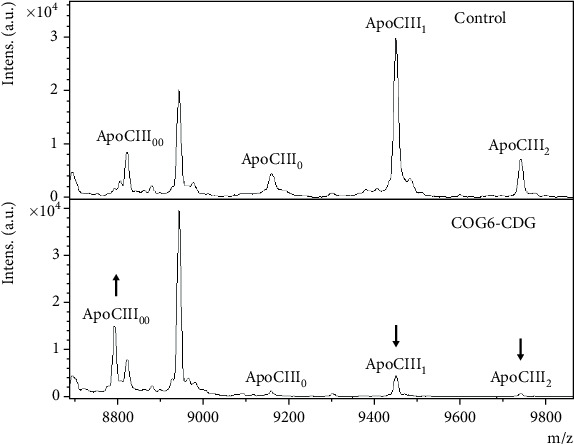
O-glycoprofile of apolipoprotein CIII (ApoCIII) determined by MALDI-TOF MS. In COG6-CDG, increased levels of ApoCIII with an unoccupied O-glycosylation site, altogether with decreased levels of both of its sialylated glycoisoforms, were detected (depicted by black arrows). ApoCIII glycoisoforms: _00_-aglycosylated; _0_-asialylated (HexNAc1Hex1); _1_-monosialylated (HexNAc1Hex1NeuAc1); _2_-disialylated (HexNAc1Hex1NeuAc2). Hex, hexose; HexNAc, *N*-acetylglucosamine; NeuAc, sialic acid.

Analysis of ApoCIII O-glycoisoforms in the patient's sample revealed significantly increased relative levels of aglycosylated ApoCIII (70.0%) complemented by decreased representation of asialylated (6.2%), monosialylated (22.6%), and disialylated (4.3%) ApoCIII (Figure 6). Furthermore, the total serum O-glycoprofile analysis revealed an abnormal O-glycosylation of serum proteins represented by decreased levels of all Hex1-2HexNAc1-2NeuAc1-2 structures (Figure S7).

## 4. Discussion

The most frequent features in the previously reported COG6-CDG patients involve growth retardation, developmental disability, microcephaly, liver and gastrointestinal disease, recurrent infections, and hypohidrosis/hyperthermia [2, 7], which align with the lethal infant phenotype of COG6-CDG (OMIM #606977) in the presented patient. The most severe COG6-CDG phenotype with early lethality is more frequently associated with loss-of-function variants [7, 13, 15]. The list of previously reported COG6-CDG patients, including the index patient of this study, is summarized in Table S4.

The COG6 subunit of the COG complex is essential for maintaining the normal structure and activity of the Golgi apparatus. Malfunctions in the COG complex significantly impact processes such as protein sorting, glycosylation, and Golgi integrity [33]. In addition to the absence of COG6 protein in the patient, immunofluorescence microscopy confirmed that patient cells exhibited a delay in retrograde transport from the Golgi apparatus to the ER. Earlier studies showed that the absence of some COG subunits and COG protein instability could be linked to reductions in one or many other COG subunits [34, 35]. In particular, COG6 depletion can cause instability of the other Lobe B COG subunits (including COG5, COG7, and COG8) in both COG6-CDG patients and cell lines [6, 10, 19, 36, 37]. In our study, the reduced levels of COG7 and COG1 were observed, whereas the levels of COG4 protein, located in Lobe A, were not significantly altered in the patient's fibroblasts. However, the levels of other COG subunits were not determined. Interestingly, overexpression of LAMP2, a major marker of the lysosomal compartment, was observed. The EL (endosome-lysosome) system is involved in the turnover of damaged proteins and structural reorganization in response to changing conditions [38]. Upregulated LAMP proteins suggest an increasing amount of lysosomes [39]. The observed increases in EL components likely represent adaptive mechanisms to restore metabolic and structural abnormalities that follow COG6 defect or possible problems in trafficking in the *trans*-Golgi network [40]. Furthermore, as COG complex subunits are required for double-membrane cytoplasm to vacuole targeting vesicle and autophagosome formation [41], the inhibition of autophagy flux can also explain LAMP2 accumulation. In addition, its overexpression can be related to COG defect–related vacuolization since the generation of LAMP2-positive enlarged endolysosomal vacuoles was confirmed in COG KOs as well as in COG7-deficient fibroblasts [42]. Furthermore, the observed slight shift in LAMP2 migration is consistent with altered N-glycosylation in CDG patients.

Based on our knowledge, the analysis resulted in a Type II pattern in all but one case of COG6-CDG where a Tf glycosylation was analyzed. However, in Shaheen syndrome, none of the patients showed detectable abnormalities in the glycosylation of Tf [17]. In addition to an abnormal Tf N-glycoprofile found in the sample of the presented patient, most of the ApoCIII molecules were not occupied by any O-glycan, whereas in controls, the aglycosylated ApoCIII accounted for less than 12% (internal reference range). An increase in underglycosylated and unglycosylated structures, consistent with a mucin-type Core 1 O-glycosylation defect, was described in another COG6-CDG patient [10]; however, the levels of individual glycoisoforms were not quantified in this study.

The significant reduction in both ApoCIII and the total serum O-glycoprofile was observed for the monosialylated and disialylated T-antigen, which are formed by the addition of sialic acid to HexNAc1Hex1 disaccharide, known as the Core 1 T-antigen. The level of asialylated Core 1 T-antigen was not determined in the total serum O-glycoprofile due to its relatively low molecular weight, which falls within the area where matrix ion interference and detector saturation are unavoidable [43]. However, the analysis of intact ApoCIII with its glycoisoforms enabled the identification of a significant underoccupation of its O-glycosylation site. The decrease in higher Hex2HexNAc2NeuAc1-2 Core 2 O-glycans in serum, formed later by further extensions and terminations, was less prominent. These results suggest the most intense effect of the presented *COG6* mutation at the beginning of the O-glycosylation pathway, starting in *cis*-Golgi stacks. Based on our knowledge, the plasma total O-glycoprofile of COG6-CDG patients has not yet been described.

A previously reported COG6-CDG patient has shown a severely altered serum N-glycan profile with increased amounts of unprocessed or improperly processed structures such as oligomannose and truncated complex glycans lacking sialic acid, galactose, and/or *N*-acetylglucosamine [10]. Moreover, significantly increased levels of all the fucosylated species were reported in this study. Our results from the MALDI-TOF analysis of serum N-glycoprofile did not show a considerable increase in overall fucosylation. However, the increased amounts of underprocessed N-glycans were clearly confirmed by both MALDI and ESI MS–based approaches as well as HPLC-FLD analysis. The sum of relative intensities of oligomannose N-glycans, determined by MALDI-TOF, was 23% in the COG6-CDG patient and 7% in the control serum. Almost all the individual oligomannose glycans were detected in increased levels in the patient's sample, including Hex3HexNAc2 and Hex4HexNAc2 (both accounted for 0.2%), that are considered glycobiomarkers for PMM2-CDG [44] and were not detected in the control. The results from N-glycoprofiling of lysed fibroblasts confirmed increased levels of truncated and oligomannose glycans and decreased levels of sialylation.

However, the most significant changes in serum concerned the relative levels of underprocessed Hex3HexNAc3-4, Hex4HexNAc3-4, and their sialylated Hex4HexNAc3-4NeuAc1 structures. Based on the results from the MALDI-TOF analysis, the level of Hex3HexNAc3 was increased by almost 22-fold in the COG6-CDG patient compared with the control. This structure is formed in the *cis*-Golgi and further transferred to the *medial*-Golgi, where a single HexNAc unit is added to form Hex3HexNAc4 [45], the levels of which were increased almost 16-fold in the patient's serum. Thus, the obtained data suggest that N-glycan processing and maturation are mainly impacted as early as at their start in the Golgi. Significant accumulation of Hex3HexNAc3-4 in COG6-CDG suggests the probable disruption of glycan processing in *cis*-/*medial*-Golgi. Additionally, the levels of completed galactosylated and sialylated N-glycans, formed later in the *medial*- and *trans*-Golgi, were also decreased in the COG6-CDG sample; however, these decreases were not as significant as the alterations in the levels of unprocessed Hex3-4HexNAc3-4-derived structures. Increased levels of Hex3HexNAc4 in COG6-CDG were described previously [6]. However, no MS investigations of COG6-CDG patients have yet revealed increased levels of Hex3HexNAc3 N-glycan. Our results confirmed this structure significantly increased in both the patient's isolated Tf and whole serum.

Based on our knowledge, only three publications have investigated the glycoprofile of COG6-CDG patients by MS. The first work by using HPLC-FLD and MALDI-TOF analysis demonstrated that the N-linked oligosaccharides on the patient's Tf have a complete or partial loss of galactose and neuraminic acid residues, represented by increased levels of Hex3-4HexNAc4, Hex4HexNAc4NeuAc1, and Hex5HexNAc4NeuAc0-1 [6], which was confirmed in our study. The second work by Huybrechts et al. focused on MALDI-TOF analysis of permethylated acidic N-glycans of Tf, establishing an apparent increase in the biantennary monosialylated N-glycan and a slight increase in Hex4HexNAc4NeuAc1 [11], which is in full agreement with our results. However, neutral structures were not analyzed. The third recent study [10] utilized the MALDI-TOF analysis of permethylated serum N-glycome. Remarkable alterations such as severe hyposialylation and hypogalactosylation, together with the occurrence of abnormal structures lacking antennary *N*-acetylglucosamine, significantly increased levels of oligomannose structures and all the fucosylated species were determined in this study; however, as in the case of ApoCIII, the levels of individual glycoisoforms were not precisely quantified. Thus, we provided comprehensive characterization of the N-glycoprofile of serum from the COG6-CDG patient, including the precise relative quantification of individual structures by two independent methods (Tables S1 and S3). Even if each technique led to slightly different values, both confirmed increased levels of presented structures, strongly supporting our claims (Table 1). In this study, data from both ESI-Orbitrap and MALDI-TOF are presented to demonstrate consistent glycan profiling trends, confirming findings across platforms. ESI-Orbitrap offers higher resolution and sensitivity, whereas MALDI-TOF enables more rapid and accessible screening in clinical biochemistry laboratories. However, since only one individual patient was involved in this study, the specificity and reliability of these potential biomarkers should be evaluated on a larger sample set.

The results obtained from clinical, biochemical, genomics, and glycomics areas jointly confirmed the defect in COG-mediated Golgi trafficking in the index patient, leading to severe phenotype and early lethality. Most rare diseases are very severe, chronic, and life-threatening and have not yet been well characterized [46]. Therefore, identifying potential COG6-CDG glycobiomarkers is crucial for early diagnosis, which continues to represent a major bottleneck in rare disease management.

## 5. Conclusion

This study focused on the clinical, biochemical, and multiomics characterization of the first COG6-CDG patient in Slovakia. Using advanced MS-based approaches, we identified a set of potential N-glycan biomarkers that may serve as a foundation for early and accurate diagnostics. Since these results are based on a single patient, the observed alterations can only be regarded as potential biomarkers and require further validation. Collaboration among clinical laboratories, pediatricians, geneticists, and scientists is essential for a better understanding of the molecular basis of such disorders, particularly when previously unidentified genetic variants are involved.

## Figures and Tables

**Table 1:** Representative relative abundances of potential COG6-CDG glycobiomarkers in the patient's serum and control, determined by two independent methods. MALDI-TOF analysis of permethylated glycans and ESI-Orbitrap analysis of RapiFluor-MS–labeled glycans were utilized to determine the relative levels of individual structures.

	**Permethylated N-glycans**		**RapiFluor-MS–labeled N-glycans**	
	**MALDI-TOF**		**ESI-Orbitrap**	
	**COG6-CDG**	**Control**	**COG6-CDG**	**Control**
Hex3HexNAc2	0.17%	0.00%	1.34%	0.00%
Hex4HexNAc2	0.22%	0.00%	0.74%	0.14%
Hex5HexNAc2	11.23%	1.80%	4.16%	0.97%
Hex3HexNAc3	2.17%	0.10%	4.38%	0.00%
Hex4HexNAc3	0.91%	0.15%	0.53%	0.00%
Hex4HexNAc3NeuAc1	2.26%	0.24%	4.19%	0.46%
Hex5HexNAc3	0.95%	0.31%	0.25%	0.00%
Hex3HexNAc4	2.64%	0.17%	1.77%	0.14%
Hex4HexNAc4	2.95%	0.54%	1.04%	0.20%
Hex4HexNAc4NeuAc1	3.64%	0.39%	8.70%	0.36%

## Data Availability

The data that support the findings of this study are openly available in GlycoPOST at https://glycopost.glycosmos.org/, Reference Number GPST000459.

## Supporting Information

Supporting Information 1 Figure S1: Amino acid and DNA sequences of COG6 of both wild type and presented patient with depicted positions of mutation. This figure shows the following: (i) the wild type coding DNA sequence of *COG6* gene (1974 nucleotides) with depicted nucleotides at positions 906 and 907 that are deleted in the patient's DNA; (ii) original amino acid sequence of COG6 protein (657 amino acids) with depicted amino acid (His at the position 302), which is mutated; (iii) mutated coding DNA sequence of COG6 gene (1973 nucleotides) with depicted nucleotide at the position 906 where A is inserted; and (iv) mutated COG6 protein consisting of only 304 amino acids. Furthermore, the translation of both wild type and mutated DNA into amino acid sequences, DNA alignment of both wild type and mutated nucleotide sequence, and protein alignment of both wild type and mutated truncated amino acid sequence are shown in this file.

Supporting Information 2 Figure S2: Relative distribution of transferrin N-glycan structures in the sample of COG6-CDG patient and control. Data are expressed as average ± SD of two replicates. Fuc, fucose; Hex, hexose; HexNAc, *N*-acetylglucosamine; NeuAc, sialic acid.

Supporting Information 3 Figure S3: Representative MS/MS (A) and MS spectrum (B) of Hex3HexNAc3 N-glycan (*m/z* 1416.7), whose relative levels were increased by almost 22-fold in COG6-CDG serum when compared with controls, based on MALDI-TOF analysis. Hex, hexose (green circle); HexNAc, *N*-acetylhexosamine (blue square).

Supporting Information 4 Figure S4: MALDI-TOF spectra of permethylated N-glycans from sera of COG6-CDG patient and negative control.

Supporting Information 5 Figure S5: MALDI-TOF spectra of permethylated N-glycans from fibroblasts of COG6-CDG patient and control.

Supporting Information 6 Figure S6: Representative EICs of Hex3HexNAc3 and Hex3HexNAc4 N-glycans of both COG6-CDG patient and control.

Supporting Information 7 Figure S7: Relative intensities of individual serum O-glycans of both COG6-CDG patient and control. Data are expressed as average ± SEM of two replicates. Hex, hexose; HexNAc, *N*-acetylhexosamine; NeuAc—sialic acid.

Supporting Information 8 Table S1: Data obtained by MALDI-TOF analysis of serum N-glycoprofile of COG6-CDG patient and control.

Supporting Information 9 Table S2: Data obtained by MALDI-TOF analysis of permethylated N-glycans from fibroblasts of COG6-CDG patient and control.

Supporting Information 10 Table S3: Data obtained by ESI-Orbitrap analysis of serum N-glycoprofile of COG6-CDG patient and control.

Supporting Information 11 Table S4: The list of previously reported COG6-CDG patients, including the index patient of this study.
