# Supplementary material for: Insights Into the Pathological Glycosylation Associated With COG6-CDG
Source: Hum Mutat. 2025 Nov 30;2025:7948771. doi: 10.1155/humu/7948771 (PMC12682456; doi:10.1155/humu/7948771)
Supplement: Supporting Information 1 — Figure S1: Amino acid and DNA sequences of COG6 of both wild type and presented patient with depicted positions of mutation. This figure shows the following: (i) the wild type coding DNA sequence of COG6 gene (1974 nucleotides) with depicted nucleotides at positions 906 and 907 that are deleted in the patient's DNA; (ii) original amino acid sequence of COG6 protein (657 amino acids) with depicted amino acid (His at the position 302), which is mutated; (iii) mutated coding DNA sequence of COG6 gene (1973 nucleotides) with depicted nucleotide at the position 906 where A is inserted; and (iv) mutated COG6 protein consisting of only 304 amino acids. Furthermore, the translation of both wild type and mutated DNA into amino acid sequences, DNA alignment of both wild type and mutated nucleotide sequence, and protein alignment of both wild type and mutated truncated amino acid sequence are shown in this file. [file 7948771.f1.pdf]

>BC051723.1:51-2024 HOMO SAPIENS COMPONENT OF OLIGOMERIC GOLGI COMPLEX 6, MRNA (CDNA CLONE MGC:48438 IMAGE:5263056), COMPLETE CDS (HTTPS://WWW.NCBI.NLM.NIH.GOV/NUCCORE/BC051723.1?FROM=51&TO=2024&REPORT=FASTA) (1974 NT)
ATGGCAGAGGGCAGCGGGGAAGTGGTCGCAGTGTCTGCGACCGGGGCTGCCAACGGCCTCAACAATGGGGCAGGCGGGACCTCGGCGACGACCT
GCAACCCGCTGTCGCGCAAGCTGCATAAGATCCTGGAGACGCGGCTGGACAACGACAAGGAGATGTTAGAAGCTCTCAAGGCACTTTCAACCTT
TTTTGTTGAAAATAGTCTGCGGACTCGAAGAAATTTACGTGGAGATATTGAACGTAAAAGTTTAGCCATCAATGAAGAATTTGTAAGCATTTTC
AAGGAAGTGAAGGAGGAACTTGAAAGCATAAGCGAAGATGTTCAAGCAATGAGCAACTGTTGTCAAGATATGACAAGTCGCCTACAGGCAGCAA
AGGAACAGACTCAAGATTTAATAGTAAAAACCACTAAGCTTCAATCTGAAAGCCAAAAATTAGAGATAAGAGCTCAAGTTGCAGATGCCTTCTT
ATCCAAGTTCCAACTGACTTCTGATGAAATGAGTCTTCTCCGAGGTACAAGAGAAGGACCCATTACTGAGGATTTTTTCAAGGCACTGGGAAGA
GTAAAACAGATTCATAATGATGTCAAAGTTCTCTTGCGTACAAATCAACAAACGGCAGGTTTAGAAATTATGGAACAGATGGCCTTACTTCAAG
AAACGGCTTATGAAAGACTTTACCGATGGGCTCAAAGTGAATGCAGAACATTGACACAAGAATCATGTGACGTATCTCCGGTATTGACACAGGC
AATGGAAGCCCTGCAGGACAGACCTGTCTTATATAAATATACCTTAGATGAATTTGGAACAGCCAGAAGAAGTACAGTTGTTCGTGGATTTATT
GATGCGCTCACAAGAGGGGGCCCCGGAGGTACACCTAGACCAATTGAAATGCATTCTCATGACCCTTTGAGGTATGTAGGAGATATGTTGGCTT
GGCTCCATCAAGCTACTGCTTCTGAAAAGGAACACCTTGAAGCTCTCTTAAAGCATGTAACTACACAAGGTGTTGAAGAAAATATTCAAGAAGT
TGTTGGGCATATCACTGAAGGTGTGTGCAGGCCTCTAAAGGTTCGAATTGAGCAAGTAATAGTTGCTGAACCTGGGGCAGTTTTATTATATAAA
ATTTCTAATCTCCTCAAATTTTATCACCATACAATCAGTGGTATTGTTGGAAATAGTGCAACTGCATTATTGACTACCATTGAAGAAATGCATT
TGCTAAGCAAAAAAATATTCTTCAATAGCTTGAGTCTTCATGCAAGTAAATTAATGGACAAGGTTGAACTCCCACCACCTGATCTTGGACCAAG
TTCTGCACTAAATCAGACACTCATGTTGCTGCGTGAAGTTTTAGCATCTCACGATTCTTCAGTTGTACCATTAGATGCTCGTCAAGCTGATTTT
GTGCAGGTTTTATCATGTGTCTTGGATCCTCTCCTACAGATGTGTACTGTATCAGCCAGCAATTTAGGCACAGCTGACATGGCCACTTTCATGG
TCAATTCACTATATATGATGAAGACAACATTAGCTCTATTTGAATTCACTGACAGACGTCTGGAAATGCTACAGTTTCAGATCGAAGCACATTT
GGACACACTTATAAATGAGCAAGCCTCTTATGTTTTAACTAGGGTAGGCTTGAGTTACATCTATAACACTGTACAGCAACATAAACCTGAACAG
GGCTCTTTAGCTAATATGCCCAACCTAGATTCTGTGACACTGAAGGCTGCAATGGTTCAGTTTGATCGTTATCTGTCAGCCCCAGACAACCTAT
TGATACCACAGCTGAACTTTCTTCTAAGTGCCACAGTGAAAGAGCAGATCGTAAAACAATCTACAGAATTAGTCTGCAGAGCCTATGGTGAAGT
GTATGCAGCCGTGATGAATCCAATCAATGAATACAAAGATCCAGAGAACATTCTTCACCGATCGCCGCAGCAAGTGCAGACGCTTCTTTCCTGA

>SP|Q9Y2V7|COG6_HUMAN CONSERVED OLIGOMERIC GOLGI COMPLEX SUBUNIT 6 OS=HOMO SAPIENS OX=9606 GN=COG6 PE=1 SV=2 (HTTPS://WWW.UNIPROT.ORG/UNIPROTKB/Q9Y2V7/ENTRY#SEQUENCES) (657 AA)
MAEGSGEVVAVSATGAANGLNNGAGGTSATTCNPLSRKLHKILETRLDNDKEMLEALKALSTFFVENSLRTRRNLRGDIERKSLAINEEFVSIF
KEVKEELESISEDVQAMSNCCQDMTSRLQAAKEQTQDLIVKTTKLQSESQKLEIRAQVADAFLSKFQLTSDEMSLLRGTREGPITEDFFKALGR
VKQIHNDVKVLLRTNQQTAGLEIMEQMALLQETAYERLYRWAQSECRTLTQESCDVSPVLTQAMEALQDRPVLYKYTLDEFGTARRSTVVRGFI
DALTRGGPGGTPRPIEMHSHDPLRYVGDMLAWLHQATASEKEHLEALLKHVTTQGVEENIQEVVGHITEGVCRPLKVRIEQVIVAEPGAVLLYK
ISNLLKFYHHTISGIVGNSATALLTTIEEMHLLSKKIFFNSLSLHASKLMDKVELPPPDLGPSSALNQTLMLLREVLASHDSSVVPLDARQADF
VQVLSCVLDPLLQMCTVSASNLGTADMATFMVNSLYMMKTTLALFEFTDRRLEMLQFQIEAHLDTLINEQASYVLTRVGLSYIYNTVQQHKPEQ
GSLANMPNLDSVTLKAAMVQFDRYLSAPDNLLIPQLNFLLSATVKEQIVKQSTELVCRAYGEVYAAVMNPINEYKDPENILHRSPQQVQTLLS

>MUT DNA (1973 NT)
ATGGCAGAGGGCAGCGGGGAAGTGGTCGCAGTGTCTGCGACCGGGGCTGCCAACGGCCTCAACAATGGGGCAGGCGGGACCTCGGCGACGACCT
GCAACCCGCTGTCGCGCAAGCTGCATAAGATCCTGGAGACGCGGCTGGACAACGACAAGGAGATGTTAGAAGCTCTCAAGGCACTTTCAACCTT
TTTTGTTGAAAATAGTCTGCGGACTCGAAGAAATTTACGTGGAGATATTGAACGTAAAAGTTTAGCCATCAATGAAGAATTTGTAAGCATTTTC
AAGGAAGTGAAGGAGGAACTTGAAAGCATAAGCGAAGATGTTCAAGCAATGAGCAACTGTTGTCAAGATATGACAAGTCGCCTACAGGCAGCAA
AGGAACAGACTCAAGATTTAATAGTAAAAACCACTAAGCTTCAATCTGAAAGCCAAAAATTAGAGATAAGAGCTCAAGTTGCAGATGCCTTCTT
ATCCAAGTTCCAACTGACTTCTGATGAAATGAGTCTTCTCCGAGGTACAAGAGAAGGACCCATTACTGAGGATTTTTTCAAGGCACTGGGAAGA
GTAAAACAGATTCATAATGATGTCAAAGTTCTCTTGCGTACAAATCAACAAACGGCAGGTTTAGAAATTATGGAACAGATGGCCTTACTTCAAG
AAACGGCTTATGAAAGACTTTACCGATGGGCTCAAAGTGAATGCAGAACATTGACACAAGAATCATGTGACGTATCTCCGGTATTGACACAGGC
AATGGAAGCCCTGCAGGACAGACCTGTCTTATATAAATATACCTTAGATGAATTTGGAACAGCCAGAAGAAGTACAGTTGTTCGTGGATTTATT
GATGCGCTCACAAGAGGGGGCCCCGGAGGTACACCTAGACCAATTGAAATGCATTCTCAAACCCTTTGAGGTATGTAGGAGATATGTTGGCTTG
GCTCCATCAAGCTACTGCTTCTGAAAAGGAACACCTTGAAGCTCTCTTAAAGCATGTAACTACACAAGGTGTTGAAGAAAATATTCAAGAAGTT
GTTGGGCATATCACTGAAGGTGTGTGCAGGCCTCTAAAGGTTCGAATTGAGCAAGTAATAGTTGCTGAACCTGGGGCAGTTTTATTATATAAAA
TTTCTAATCTCCTCAAATTTTATCACCATACAATCAGTGGTATTGTTGGAAATAGTGCAACTGCATTATTGACTACCATTGAAGAAATGCATTT
GCTAAGCAAAAAAATATTCTTCAATAGCTTGAGTCTTCATGCAAGTAAATTAATGGACAAGGTTGAACTCCCACCACCTGATCTTGGACCAAGT
TCTGCACTAAATCAGACACTCATGTTGCTGCGTGAAGTTTTAGCATCTCACGATTCTTCAGTTGTACCATTAGATGCTCGTCAAGCTGATTTTG
TGCAGGTTTTATCATGTGTCTTGGATCCTCTCCTACAGATGTGTACTGTATCAGCCAGCAATTTAGGCACAGCTGACATGGCCACTTTCATGGT
CAATTCACTATATATGATGAAGACAACATTAGCTCTATTTGAATTCACTGACAGACGTCTGGAAATGCTACAGTTTCAGATCGAAGCACATTTG
GACACACTTATAAATGAGCAAGCCTCTTATGTTTTAACTAGGGTAGGCTTGAGTTACATCTATAACACTGTACAGCAACATAAACCTGAACAGG
GCTCTTTAGCTAATATGCCCAACCTAGATTCTGTGACACTGAAGGCTGCAATGGTTCAGTTTGATCGTTATCTGTCAGCCCCAGACAACCTATT
GATACCACAGCTGAACTTTCTTCTAAGTGCCACAGTGAAAGAGCAGATCGTAAAACAATCTACAGAATTAGTCTGCAGAGCCTATGGTGAAGTG
TATGCAGCCGTGATGAATCCAATCAATGAATACAAAGATCCAGAGAACATTCTTCACCGATCGCCGCAGCAAGTGCAGACGCTTCTTTCCTGA

>MUT PROT (304 AA)
MAEGSGEVVAVSATGAANGLNNGAGGTSATTCNPLSRKLHKILETRLDNDKEMLEALKALSTFFVENSLRTRRNLRGDIERKSLAINEEFVSIF
KEVKEELESISEDVQAMSNCCQDMTSRLQAAKEQTQDLIVKTTKLQSESQKLEIRAQVADAFLSKFQLTSDEMSLLRGTREGPITEDFFKALGR
VKQIHNDVKVLLRTNQQTAGLEIMEQMALLQETAYERLYRWAQSECRTLTQESCDVSPVLTQAMEALQDRPVLYKYTLDEFGTARRSTVVRGFI
DALTRGGPGGTPRPIEMHSQTL

>WILD TYPE TRANSLATION
ATG GCA GAG GGC AGC GGG GAA GTG GTC GCA GTG TCT GCG ACC GGG GCT GCC AAC GGC CTC
M A E G S G E V V A V S A T G A A N G L
AAC AAT GGG GCA GGC GGG ACC TCG GCG ACG ACC TGC AAC CCG CTG TCG CGC AAG CTG CAT
N N G A G G T S A T T C N P L S R K L H
AAG ATC CTG GAG ACG CGG CTG GAC AAC GAC AAG GAG ATG TTA GAA GCT CTC AAG GCA CTT
K I L E T R L D N D K E M L E A L K A L
TCA ACC TTT TTT GTT GAA AAT AGT CTG CGG ACT CGA AGA AAT TTA CGT GGA GAT ATT GAA
S T F F V E N S L R T R R N L R G D I E
CGT AAA AGT TTA GCC ATC AAT GAA GAA TTT GTA AGC ATT TTC AAG GAA GTG AAG GAG GAA
R K S L A I N E E F V S I F K E V K E E
CTT GAA AGC ATA AGC GAA GAT GTT CAA GCA ATG AGC AAC TGT TGT CAA GAT ATG ACA AGT
L E S I S E D V Q A M S N C C Q D M T S
CGC CTA CAG GCA GCA AAG GAA CAG ACT CAA GAT TTA ATA GTA AAA ACC ACT AAG CTT CAA
R L Q A A K E Q T Q D L I V K T T K L Q
TCT GAA AGC CAA AAA TTA GAG ATA AGA GCT CAA GTT GCA GAT GCC TTC TTA TCC AAG TTC
S E S Q K L E I R A Q V A D A F L S K F
CAA CTG ACT TCT GAT GAA ATG AGT CTT CTC CGA GGT ACA AGA GAA GGA CCC ATT ACT GAG
Q L T S D E M S L L R G T R E G P I T E
GAT TTT TTC AAG GCA CTG GGA AGA GTA AAA CAG ATT CAT AAT GAT GTC AAA GTT CTC TTG
D F F K A L G R V K Q I H N D V K V L L
CGT ACA AAT CAA CAA ACG GCA GGT TTA GAA ATT ATG GAA CAG ATG GCC TTA CTT CAA GAA
R T N Q Q T A G L E I M E Q M A L L Q E
ACG GCT TAT GAA AGA CTT TAC CGA TGG GCT CAA AGT GAA TGC AGA ACA TTG ACA CAA GAA
T A Y E R L Y R W A Q S E C R T L T Q E
TCA TGT GAC GTA TCT CCG GTA TTG ACA CAG GCA ATG GAA GCC CTG CAG GAC AGA CCT GTC
S C D V S P V L T Q A M E A L Q D R P V
TTA TAT AAA TAT ACC TTA GAT GAA TTT GGA ACA GCC AGA AGA AGT ACA GTT GTT CGT GGA
L Y K Y T L D E F G T A R R S T V V R G
TTT ATT GAT GCG CTC ACA AGA GGG GGC CCC GGA GGT ACA CCT AGA CCA ATT GAA ATG CAT
F I D A L T R G G P G G T P R P I E M H
TCT CAT GAC CCT TTG AGG TAT GTA GGA GAT ATG TTG GCT TGG CTC CAT CAA GCT ACT GCT
S H D P L R Y V G D M L A W L H Q A T A
TCT GAA AAG GAA CAC CTT GAA GCT CTC TTA AAG CAT GTA ACT ACA CAA GGT GTT GAA GAA
S E K E H L E A L L K H V T T Q G V E E
AAT ATT CAA GAA GTT GTT GGG CAT ATC ACT GAA GGT GTG TGC AGG CCT CTA AAG GTT CGA
N I Q E V V G H I T E G V C R P L K V R
ATT GAG CAA GTA ATA GTT GCT GAA CCT GGG GCA GTT TTA TTA TAT AAA ATT TCT AAT CTC
I E Q V I V A E P G A V L L Y K I S N L
CTC AAA TTT TAT CAC CAT ACA ATC AGT GGT ATT GTT GGA AAT AGT GCA ACT GCA TTA TTG
L K F Y H H T I S G I V G N S A T A L L
ACT ACC ATT GAA GAA ATG CAT TTG CTA AGC AAA AAA ATA TTC TTC AAT AGC TTG AGT CTT
T T I E E M H L L S K K I F F N S L S L
CAT GCA AGT AAA TTA ATG GAC AAG GTT GAA CTC CCA CCA CCT GAT CTT GGA CCA AGT TCT
H A S K L M D K V E L P P P D L G P S S
GCA CTA AAT CAG ACA CTC ATG TTG CTG CGT GAA GTT TTA GCA TCT CAC GAT TCT TCA GTT
A L N Q T L M L L R E V L A S H D S S V
GTA CCA TTA GAT GCT CGT CAA GCT GAT TTT GTG CAG GTT TTA TCA TGT GTC TTG GAT CCT
V P L D A R Q A D F V Q V L S C V L D P
CTC CTA CAG ATG TGT ACT GTA TCA GCC AGC AAT TTA GGC ACA GCT GAC ATG GCC ACT TTC
L L Q M C T V S A S N L G T A D M A T F
ATG GTC AAT TCA CTA TAT ATG ATG AAG ACA ACA TTA GCT CTA TTT GAA TTC ACT GAC AGA
M V N S L Y M M K T T L A L F E F T D R
CGT CTG GAA ATG CTA CAG TTT CAG ATC GAA GCA CAT TTG GAC ACA CTT ATA AAT GAG CAA
R L E M L Q F Q I E A H L D T L I N E Q
GCC TCT TAT GTT TTA ACT AGG GTA GGC TTG AGT TAC ATC TAT AAC ACT GTA CAG CAA CAT
A S Y V L T R V G L S Y I Y N T V Q Q H
AAA CCT GAA CAG GGC TCT TTA GCT AAT ATG CCC AAC CTA GAT TCT GTG ACA CTG AAG GCT
K P E Q G S L A N M P N L D S V T L K A
GCA ATG GTT CAG TTT GAT CGT TAT CTG TCA GCC CCA GAC AAC CTA TTG ATA CCA CAG CTG
A M V Q F D R Y L S A P D N L L I P Q L
AAC TTT CTT CTA AGT GCC ACA GTG AAA GAG CAG ATC GTA AAA CAA TCT ACA GAA TTA GTC
N F L L S A T V K E Q I V K Q S T E L V
TGC AGA GCC TAT GGT GAA GTG TAT GCA GCC GTG ATG AAT CCA ATC AAT GAA TAC AAA GAT
C R A Y G E V Y A A V M N P I N E Y K D
CCA GAG AAC ATT CTT CAC CGA TCG CCG CAG CAA GTG CAG ACG CTT CTT TCC TGA
P E N I L H R S P Q Q V Q T L L S -

>MUTATED TRANSLATION
ATG GCA GAG GGC AGC GGG GAA GTG GTC GCA GTG TCT GCG ACC GGG GCT GCC AAC GGC CTC
M A E G S G E V V A V S A T G A A N G L
AAC AAT GGG GCA GGC GGG ACC TCG GCG ACG ACC TGC AAC CCG CTG TCG CGC AAG CTG CAT
N N G A G G T S A T T C N P L S R K L H
AAG ATC CTG GAG ACG CGG CTG GAC AAC GAC AAG GAG ATG TTA GAA GCT CTC AAG GCA CTT
K I L E T R L D N D K E M L E A L K A L
TCA ACC TTT TTT GTT GAA AAT AGT CTG CGG ACT CGA AGA AAT TTA CGT GGA GAT ATT GAA
S T F F V E N S L R T R R N L R G D I E
CGT AAA AGT TTA GCC ATC AAT GAA GAA TTT GTA AGC ATT TTC AAG GAA GTG AAG GAG GAA
R K S L A I N E E F V S I F K E V K E E
CTT GAA AGC ATA AGC GAA GAT GTT CAA GCA ATG AGC AAC TGT TGT CAA GAT ATG ACA AGT
L E S I S E D V Q A M S N C C Q D M T S
CGC CTA CAG GCA GCA AAG GAA CAG ACT CAA GAT TTA ATA GTA AAA ACC ACT AAG CTT CAA
R L Q A A K E Q T Q D L I V K T T K L Q
TCT GAA AGC CAA AAA TTA GAG ATA AGA GCT CAA GTT GCA GAT GCC TTC TTA TCC AAG TTC
S E S Q K L E I R A Q V A D A F L S K F
CAA CTG ACT TCT GAT GAA ATG AGT CTT CTC CGA GGT ACA AGA GAA GGA CCC ATT ACT GAG
Q L T S D E M S L L R G T R E G P I T E
GAT TTT TTC AAG GCA CTG GGA AGA GTA AAA CAG ATT CAT AAT GAT GTC AAA GTT CTC TTG
D F F K A L G R V K Q I H N D V K V L L
CGT ACA AAT CAA CAA ACG GCA GGT TTA GAA ATT ATG GAA CAG ATG GCC TTA CTT CAA GAA
R T N Q Q T A G L E I M E Q M A L L Q E
ACG GCT TAT GAA AGA CTT TAC CGA TGG GCT CAA AGT GAA TGC AGA ACA TTG ACA CAA GAA
T A Y E R L Y R W A Q S E C R T L T Q E
TCA TGT GAC GTA TCT CCG GTA TTG ACA CAG GCA ATG GAA GCC CTG CAG GAC AGA CCT GTC
S C D V S P V L T Q A M E A L Q D R P V
TTA TAT AAA TAT ACC TTA GAT GAA TTT GGA ACA GCC AGA AGA AGT ACA GTT GTT CGT GGA
L Y K Y T L D E F G T A R R S T V V R G
TTT ATT GAT GCG CTC ACA AGA GGG GGC CCC GGA GGT ACA CCT AGA CCA ATT GAA ATG CAT
F I D A L T R G G P G G T P R P I E M H
TCT CAA ACC CTT TGA GGT ATG TAG GAG ATA TGT TGG CTT GGC TCC ATC AAG CTA CTG CTT
S Q T L - G M - E I C W L G S I K L L L
CTG AAA AGG AAC ACC TTG AAG CTC TCT TAA AGC ATG TAA CTA CAC AAG GTG TTG AAG AAA
L K R N T L K L S - S M - L H K V L K K
ATA TTC AAG AAG TTG TTG GGC ATA TCA CTG AAG GTG TGT GCA GGC CTC TAA AGG TTC GAA
I F K K L L G I S L K V C A G L - R F E
TTG AGC AAG TAA TAG TTG CTG AAC CTG GGG CAG TTT TAT TAT ATA AAA TTT CTA ATC TCC
L S K - - L L N L G Q F Y Y I K F L I S
TCA AAT TTT ATC ACC ATA CAA TCA GTG GTA TTG TTG GAA ATA GTG CAA CTG CAT TAT TGA
S N F I T I Q S V V L L E I V Q L H Y -
CTA CCA TTG AAG AAA TGC ATT TGC TAA GCA AAA AAA TAT TCT TCA ATA GCT TGA GTC TTC
L P L K K C I C - A K K Y S S I A - V F
ATG CAA GTA AAT TAA TGG ACA AGG TTG AAC TCC CAC CAC CTG ATC TTG GAC CAA GTT CTG
M Q V N - W T R L N S H H L I L D Q V L
CAC TAA ATC AGA CAC TCA TGT TGC TGC GTG AAG TTT TAG CAT CTC ACG ATT CTT CAG TTG
H - I R H S C C C V K F - H L T I L Q L
TAC CAT TAG ATG CTC GTC AAG CTG ATT TTG TGC AGG TTT TAT CAT GTG TCT TGG ATC CTC
Y H - M L V K L I L C R F Y H V S W I L
TCC TAC AGA TGT GTA CTG TAT CAG CCA GCA ATT TAG GCA CAG CTG ACA TGG CCA CTT TCA
S Y R C V L Y Q P A I - A Q L T W P L S
TGG TCA ATT CAC TAT ATA TGA TGA AGA CAA CAT TAG CTC TAT TTG AAT TCA CTG ACA GAC
W S I H Y I - - R Q H - L Y L N S L T D
GTC TGG AAA TGC TAC AGT TTC AGA TCG AAG CAC ATT TGG ACA CAC TTA TAA ATG AGC AAG
V W K C Y S F R S K H I W T H L - M S K
CCT CTT ATG TTT TAA CTA GGG TAG GCT TGA GTT ACA TCT ATA ACA CTG TAC AGC AAC ATA
P L M F - L G - A - V T S I T L Y S N I
AAC CTG AAC AGG GCT CTT TAG CTA ATA TGC CCA ACC TAG ATT CTG TGA CAC TGA AGG CTG
N L N R A L - L I C P T - I L - H - R L
CAA TGG TTC AGT TTG ATC GTT ATC TGT CAG CCC CAG ACA ACC TAT TGA TAC CAC AGC TGA
Q W F S L I V I C Q P Q T T Y - Y H S -
ACT TTC TTC TAA GTG CCA CAG TGA AAG AGC AGA TCG TAA AAC AAT CTA CAG AAT TAG TCT
T F F - V P Q - K S R S - N N L Q N - S
GCA GAG CCT ATG GTG AAG TGT ATG CAG CCG TGA TGA ATC CAA TCA ATG AAT ACA AAG ATC
A E P M V K C M Q P - - I Q S M N T K I
CAG AGA ACA TTC TTC ACC GAT CGC CGC AGC AAG TGC AGA CGC TTC TTT CCT GA
Q R T F F T D R R S K C R R F F P

```
DNA ALIGNMENT
BC051723.1_51-2024      ATGGCAGAGGGCAGCGGGGAAGTGGTCGCAGTGTCTGCGACCGGGGCTGCCAACGGCCTC
MUT                     ATGGCAGAGGGCAGCGGGGAAGTGGTCGCAGTGTCTGCGACCGGGGCTGCCAACGGCCTC
                        ************************************************************

BC051723.1_51-2024      AACAATGGGGCAGGCGGGACCTCGGCGACGACCTGCAACCCGCTGTCGCGCAAGCTGCAT
MUT                     AACAATGGGGCAGGCGGGACCTCGGCGACGACCTGCAACCCGCTGTCGCGCAAGCTGCAT
                        ************************************************************

BC051723.1_51-2024      AAGATCCTGGAGACGCGGCTGGACAACGACAAGGAGATGTTAGAAGCTCTCAAGGCACTT
MUT                     AAGATCCTGGAGACGCGGCTGGACAACGACAAGGAGATGTTAGAAGCTCTCAAGGCACTT
                        ************************************************************

BC051723.1_51-2024      TCAACCTTTTTTGTTGAAAATAGTCTGCGGACTCGAAGAAATTTACGTGGAGATATTGAA
MUT                     TCAACCTTTTTTGTTGAAAATAGTCTGCGGACTCGAAGAAATTTACGTGGAGATATTGAA
                        ************************************************************

BC051723.1_51-2024      CGTAAAAGTTTAGCCATCAATGAAGAATTTGTAAGCATTTTCAAGGAAGTGAAGGAGGAA
MUT                     CGTAAAAGTTTAGCCATCAATGAAGAATTTGTAAGCATTTTCAAGGAAGTGAAGGAGGAA
                        ************************************************************

BC051723.1_51-2024      CTTGAAAGCATAAGCGAAGATGTTCAAGCAATGAGCAACTGTTGTCAAGATATGACAAGT
MUT                     CTTGAAAGCATAAGCGAAGATGTTCAAGCAATGAGCAACTGTTGTCAAGATATGACAAGT
                        ************************************************************

BC051723.1_51-2024      CGCCTACAGGCAGCAAAGGAACAGACTCAAGATTTAATAGTAAAAACCACTAAGCTTCAA
MUT                     CGCCTACAGGCAGCAAAGGAACAGACTCAAGATTTAATAGTAAAAACCACTAAGCTTCAA
                        ************************************************************

BC051723.1_51-2024      TCTGAAAGCCAAAAATTAGAGATAAGAGCTCAAGTTGCAGATGCCTTCTTATCCAAGTTC
MUT                     TCTGAAAGCCAAAAATTAGAGATAAGAGCTCAAGTTGCAGATGCCTTCTTATCCAAGTTC
                        ************************************************************

BC051723.1_51-2024      CAACTGACTTCTGATGAAATGAGTCTTCTCCGAGGTACAAGAGAAGGACCCATTACTGAG
MUT                     CAACTGACTTCTGATGAAATGAGTCTTCTCCGAGGTACAAGAGAAGGACCCATTACTGAG
                        ************************************************************

BC051723.1_51-2024      GATTTTTTCAAGGCACTGGGAAGAGTAAAACAGATTCATAATGATGTCAAAGTTCTCTTG
MUT                     GATTTTTTCAAGGCACTGGGAAGAGTAAAACAGATTCATAATGATGTCAAAGTTCTCTTG
                        ************************************************************

BC051723.1_51-2024      CGTACAAATCAACAAACGGCAGGTTTAGAAATTATGGAACAGATGGCCTTACTTCAAGAA
MUT                     CGTACAAATCAACAAACGGCAGGTTTAGAAATTATGGAACAGATGGCCTTACTTCAAGAA
                        ************************************************************

BC051723.1_51-2024      ACGGCTTATGAAAGACTTTACCGATGGGCTCAAAGTGAATGCAGAACATTGACACAAGAA
MUT                     ACGGCTTATGAAAGACTTTACCGATGGGCTCAAAGTGAATGCAGAACATTGACACAAGAA
                        ************************************************************

BC051723.1_51-2024      TCATGTGACGTATCTCCGGTATTGACACAGGCAATGGAAGCCCTGCAGGACAGACCTGTC
MUT                     TCATGTGACGTATCTCCGGTATTGACACAGGCAATGGAAGCCCTGCAGGACAGACCTGTC
                        ************************************************************

BC051723.1_51-2024      TTATATAAATATACCTTAGATGAATTTGGAACAGCCAGAAGAAGTACAGTTGTTCGTGGA
MUT                     TTATATAAATATACCTTAGATGAATTTGGAACAGCCAGAAGAAGTACAGTTGTTCGTGGA
                        ************************************************************

BC051723.1_51-2024      TTTATTGATGCGCTCACAAGAGGGGGCCCCGGAGGTACACCTAGACCAATTGAAATGCAT
MUT                     TTTATTGATGCGCTCACAAGAGGGGGCCCCGGAGGTACACCTAGACCAATTGAAATGCAT
                        ************************************************************

BC051723.1_51-2024      TCTCATGACCCTTTGAGGTATGTAGGAGATATGTTGGCTTGGCTCCATCAAGCTACTGCT
MUT                     TCTCA-AACCCTTTGAGGTATGTAGGAGATATGTTGGCTTGGCTCCATCAAGCTACTGCT
                        *****  *****************************************************

BC051723.1_51-2024      TCTGAAAAGGAACACCTTGAAGCTCTCTTAAAGCATGTAACTACACAAGGTGTTGAAGAA
MUT                     TCTGAAAAGGAACACCTTGAAGCTCTCTTAAAGCATGTAACTACACAAGGTGTTGAAGAA
                        ************************************************************

BC051723.1_51-2024      AATATTCAAGAAGTTGTTGGGCATATCACTGAAGGTGTGTGCAGGCCTCTAAAGGTTCGA
MUT                     AATATTCAAGAAGTTGTTGGGCATATCACTGAAGGTGTGTGCAGGCCTCTAAAGGTTCGA
                        ************************************************************

BC051723.1_51-2024      ATTGAGCAAGTAATAGTTGCTGAACCTGGGGCAGTTTTATTATATAAAATTTCTAATCTC
MUT                     ATTGAGCAAGTAATAGTTGCTGAACCTGGGGCAGTTTTATTATATAAAATTTCTAATCTC
                        ************************************************************
```

```
BC051723.1_51-2024      CTCAAATTTTATCACCATACAATCAGTGGTATTGTTGGAAATAGTGCAACTGCATTATTG
MUT                     CTCAAATTTTATCACCATACAATCAGTGGTATTGTTGGAAATAGTGCAACTGCATTATTG
                        ************************************************************

BC051723.1_51-2024      ACTACCATTGAAGAAATGCATTTGCTAAGCAAAAAAATATTCTTCAATAGCTTGAGTCTT
MUT                     ACTACCATTGAAGAAATGCATTTGCTAAGCAAAAAAATATTCTTCAATAGCTTGAGTCTT
                        ************************************************************

BC051723.1_51-2024      CATGCAAGTAAATTAATGGACAAGGTTGAACTCCCACCACCTGATCTTGGACCAAGTTCT
MUT                     CATGCAAGTAAATTAATGGACAAGGTTGAACTCCCACCACCTGATCTTGGACCAAGTTCT
                        ************************************************************

BC051723.1_51-2024      GCACTAAATCAGACACTCATGTTGCTGCGTGAAGTTTTAGCATCTCACGATTCTTCAGTT
MUT                     GCACTAAATCAGACACTCATGTTGCTGCGTGAAGTTTTAGCATCTCACGATTCTTCAGTT
                        ************************************************************

BC051723.1_51-2024      GTACCATTAGATGCTCGTCAAGCTGATTTTGTGCAGGTTTTATCATGTGTCTTGGATCCT
MUT                     GTACCATTAGATGCTCGTCAAGCTGATTTTGTGCAGGTTTTATCATGTGTCTTGGATCCT
                        ************************************************************

BC051723.1_51-2024      CTCCTACAGATGTGTACTGTATCAGCCAGCAATTTAGGCACAGCTGACATGGCCACTTTC
MUT                     CTCCTACAGATGTGTACTGTATCAGCCAGCAATTTAGGCACAGCTGACATGGCCACTTTC
                        ************************************************************

BC051723.1_51-2024      ATGGTCAATTCACTATATATGATGAAGACAACATTAGCTCTATTTGAATTCACTGACAGA
MUT                     ATGGTCAATTCACTATATATGATGAAGACAACATTAGCTCTATTTGAATTCACTGACAGA
                        ************************************************************

BC051723.1_51-2024      CGTCTGGAAATGCTACAGTTTCAGATCGAAGCACATTTGGACACACTTATAAATGAGCAA
MUT                     CGTCTGGAAATGCTACAGTTTCAGATCGAAGCACATTTGGACACACTTATAAATGAGCAA
                        ************************************************************

BC051723.1_51-2024      GCCTCTTATGTTTTAACTAGGGTAGGCTTGAGTTACATCTATAACACTGTACAGCAACAT
MUT                     GCCTCTTATGTTTTAACTAGGGTAGGCTTGAGTTACATCTATAACACTGTACAGCAACAT
                        ************************************************************

BC051723.1_51-2024      AAACCTGAACAGGGCTCTTTAGCTAATATGCCCAACCTAGATTCTGTGACACTGAAGGCT
MUT                     AAACCTGAACAGGGCTCTTTAGCTAATATGCCCAACCTAGATTCTGTGACACTGAAGGCT
                        ************************************************************

BC051723.1_51-2024      GCAATGGTTCAGTTTGATCGTTATCTGTCAGCCCCAGACAACCTATTGATACCACAGCTG
MUT                     GCAATGGTTCAGTTTGATCGTTATCTGTCAGCCCCAGACAACCTATTGATACCACAGCTG
                        ************************************************************

BC051723.1_51-2024      AACTTTCTTCTAAGTGCCACAGTGAAAGAGCAGATCGTAAAACAATCTACAGAATTAGTC
MUT                     AACTTTCTTCTAAGTGCCACAGTGAAAGAGCAGATCGTAAAACAATCTACAGAATTAGTC
                        ************************************************************

BC051723.1_51-2024      TGCAGAGCCTATGGTGAAGTGTATGCAGCCGTGATGAATCCAATCAATGAATACAAAGAT
MUT                     TGCAGAGCCTATGGTGAAGTGTATGCAGCCGTGATGAATCCAATCAATGAATACAAAGAT
                        ************************************************************

BC051723.1_51-2024      CCAGAGAACATTCTTCACCGATCGCCGCAGCAAGTGCAGACGCTTCTTTCCTGA
MUT                     CCAGAGAACATTCTTCACCGATCGCCGCAGCAAGTGCAGACGCTTCTTTCCTGA
                        ******************************************************
```

```
PROTEIN ALGNMENT
SP|Q9Y2V7|COG6_HUMAN      MAEGSGEVVAVSATGAANGLNNGAGGTSATTCNPLSRKLHKILETRLDNDKEMLEALKAL
MUT                       MAEGSGEVVAVSATGAANGLNNGAGGTSATTCNPLSRKLHKILETRLDNDKEMLEALKAL
                          ************************************************************

SP|Q9Y2V7|COG6_HUMAN      STFFVENSLRTRRNLRGDIERKSLAINEEFVSIFKEVKEELESISEDVQAMSNCCQDMTS
MUT                       STFFVENSLRTRRNLRGDIERKSLAINEEFVSIFKEVKEELESISEDVQAMSNCCQDMTS
                          ************************************************************

SP|Q9Y2V7|COG6_HUMAN      RLQAAKEQTQDLIVKTTKLQSESQKLEIRAQVADAFLSKFQLTSDEMSLLRGTREGPITE
MUT                       RLQAAKEQTQDLIVKTTKLQSESQKLEIRAQVADAFLSKFQLTSDEMSLLRGTREGPITE
                          ************************************************************

SP|Q9Y2V7|COG6_HUMAN      DFFKALGRVKQIHNDVKVLLRTNQQTAGLEIMEQMALLQETAYERLYRWAQSECRTLTQE
MUT                       DFFKALGRVKQIHNDVKVLLRTNQQTAGLEIMEQMALLQETAYERLYRWAQSECRTLTQE
                          ************************************************************

SP|Q9Y2V7|COG6_HUMAN      SCDVSPVLTQAMEALQDRPVLYKYTLDEFGTARRSTVVRGFIDALTRGGPGGTPRPIEMH
MUT                       SCDVSPVLTQAMEALQDRPVLYKYTLDEFGTARRSTVVRGFIDALTRGGPGGTPRPIEMH
                          ************************************************************

SP|Q9Y2V7|COG6_HUMAN      SHDPLRYVGDMLAWLHQATASEKEHLEALLKHVTTQGVEENIQEVVGHITEGVCRPLKVR
MUT                       SQTL--------------------------------------------------------
                          *:

SP|Q9Y2V7|COG6_HUMAN      IEQVIVAEPGAVLLYKISNLLKFYHHTISGIVGNSATALLTTIEEMHLLSKKIFFNSLSL
MUT                       ------------------------------------------------------------


SP|Q9Y2V7|COG6_HUMAN      HASKLMDKVELPPPDLGPSSALNQTLMLLREVLASHDSSVVPLDARQADFVQVLSCVLDP
MUT                       ------------------------------------------------------------


SP|Q9Y2V7|COG6_HUMAN      LLQMCTVSASNLGTADMATFMVNSLYMMKTTLALFEFTDRRLEMLQFQIEAHLDTLINEQ
MUT                       ------------------------------------------------------------


SP|Q9Y2V7|COG6_HUMAN      ASYVLTRVGLSYIYNTVQQHKPEQGSLANMPNLDSVTLKAAMVQFDRYLSAPDNLLIPQL
MUT                       ------------------------------------------------------------


SP|Q9Y2V7|COG6_HUMAN      NFLLSATVKEQIVKQSTELVCRAYGEVYAAVMNPINEYKDPENILHRSPQQVQTLLS
MUT                       ---------------------------------------------------------
```
