# Supplementary figures and images for: Insights Into the Pathological Glycosylation Associated With COG6-CDG
Source: Hum Mutat. 2025 Nov 30;2025:7948771. doi: 10.1155/humu/7948771 (PMC12682456; doi:10.1155/humu/7948771)

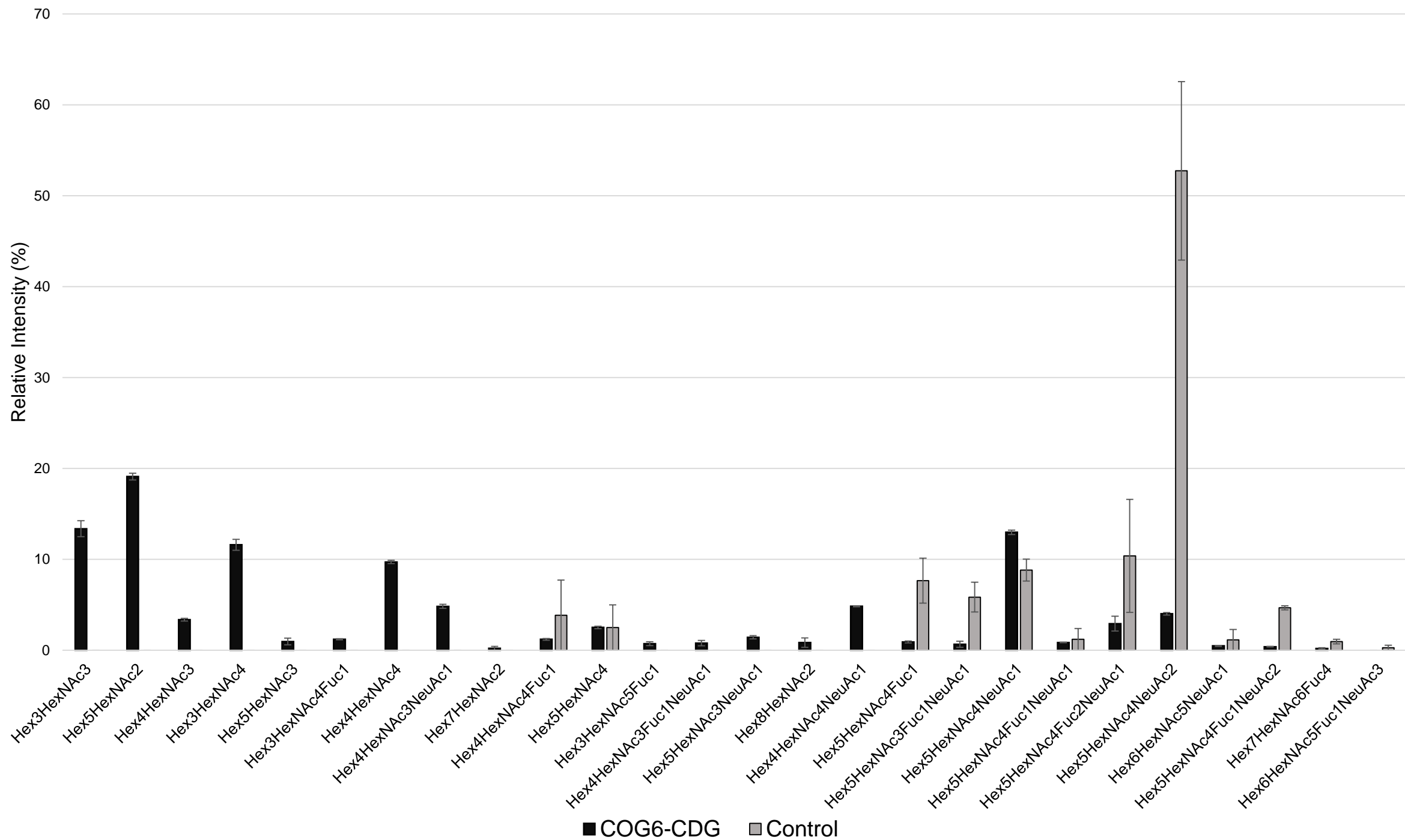

Supplement: Supporting Information 2 — Figure S2: Relative distribution of transferrin N-glycan structures in the sample of COG6-CDG patient and control. Data are expressed as average ± SD of two replicates. Fuc, fucose; Hex, hexose; HexNAc, N-acetylglucosamine; NeuAc, sialic acid. [file 7948771.f2.pdf]

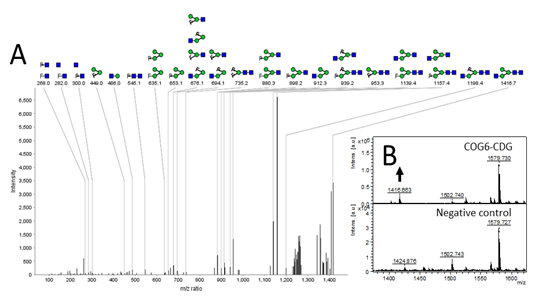

Supplement: Supporting Information 3 — Figure S3: Representative MS/MS (A) and MS spectrum (B) of Hex3HexNAc3 N-glycan (m/z 1416.7), whose relative levels were increased by almost 22-fold in COG6-CDG serum when compared with controls, based on MALDI-TOF analysis. Hex, hexose (green circle); HexNAc, N-acetylhexosamine (blue square). [file 7948771.f3.tif]

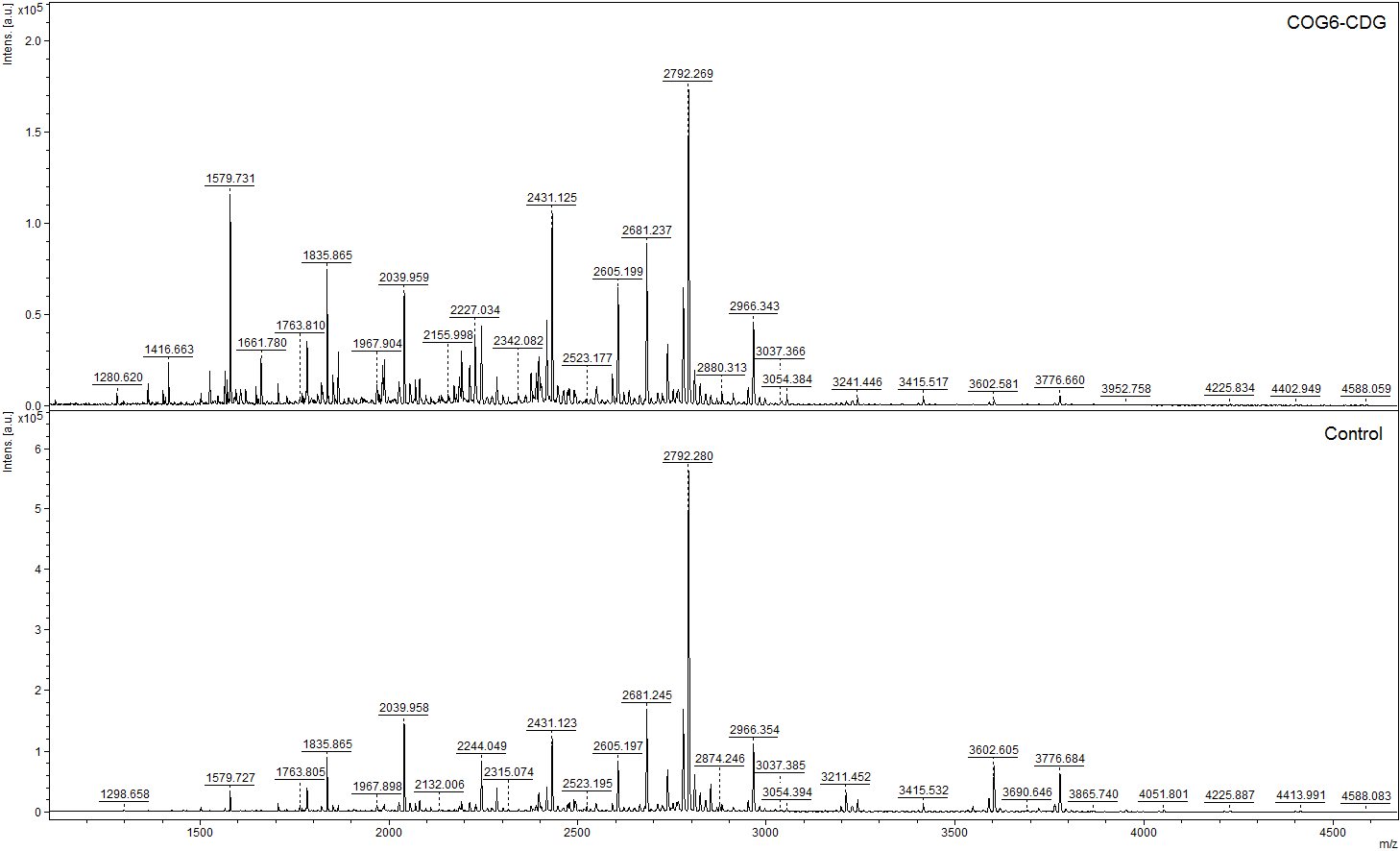

Supplement: Supporting Information 4 — Figure S4: MALDI-TOF spectra of permethylated N-glycans from sera of COG6-CDG patient and negative control. [file 7948771.f4.tif]

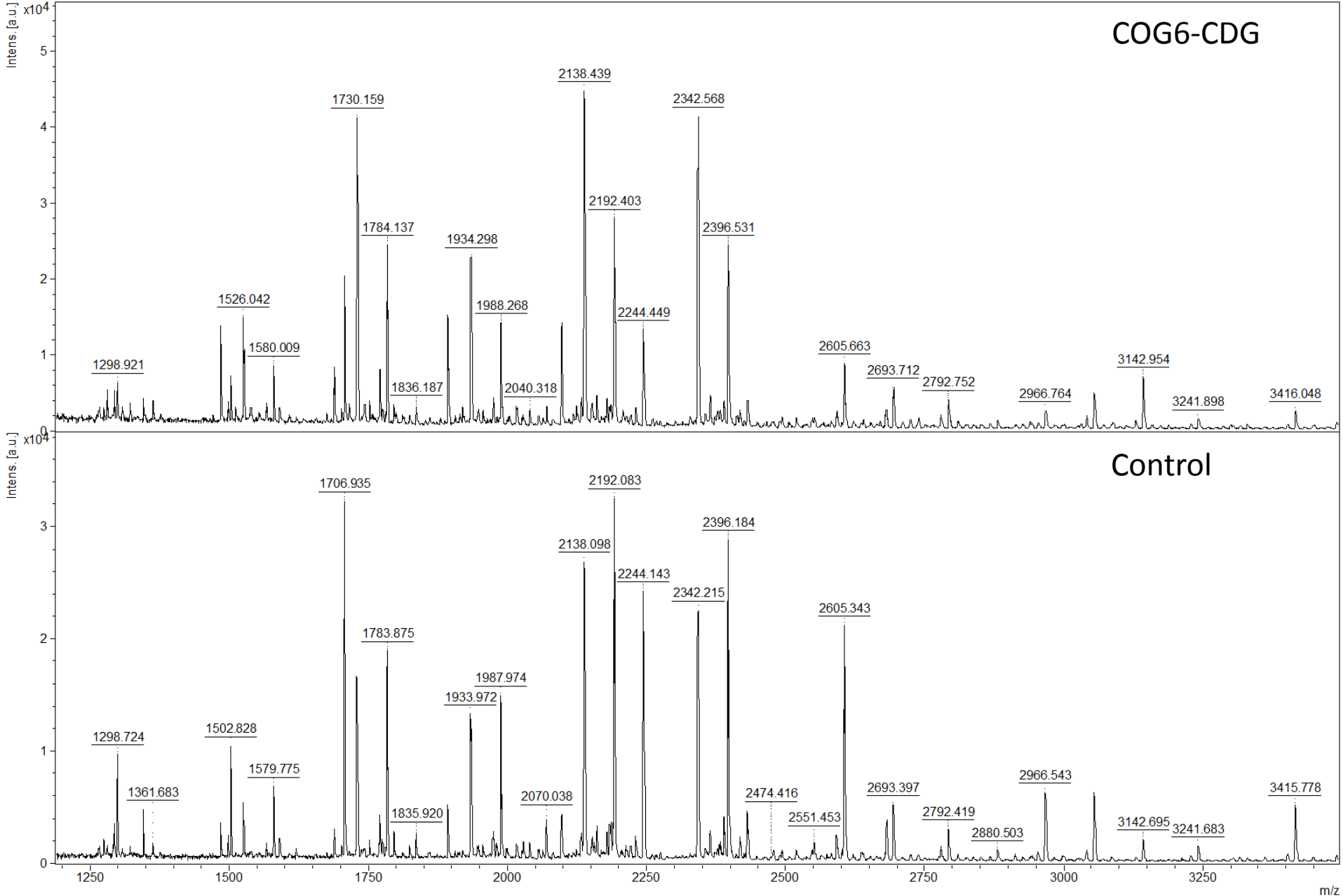

Supplement: Supporting Information 5 — Figure S5: MALDI-TOF spectra of permethylated N-glycans from fibroblasts of COG6-CDG patient and control. [file 7948771.f5.pdf]

Hex3HexNAc3 (m/z 713.3)

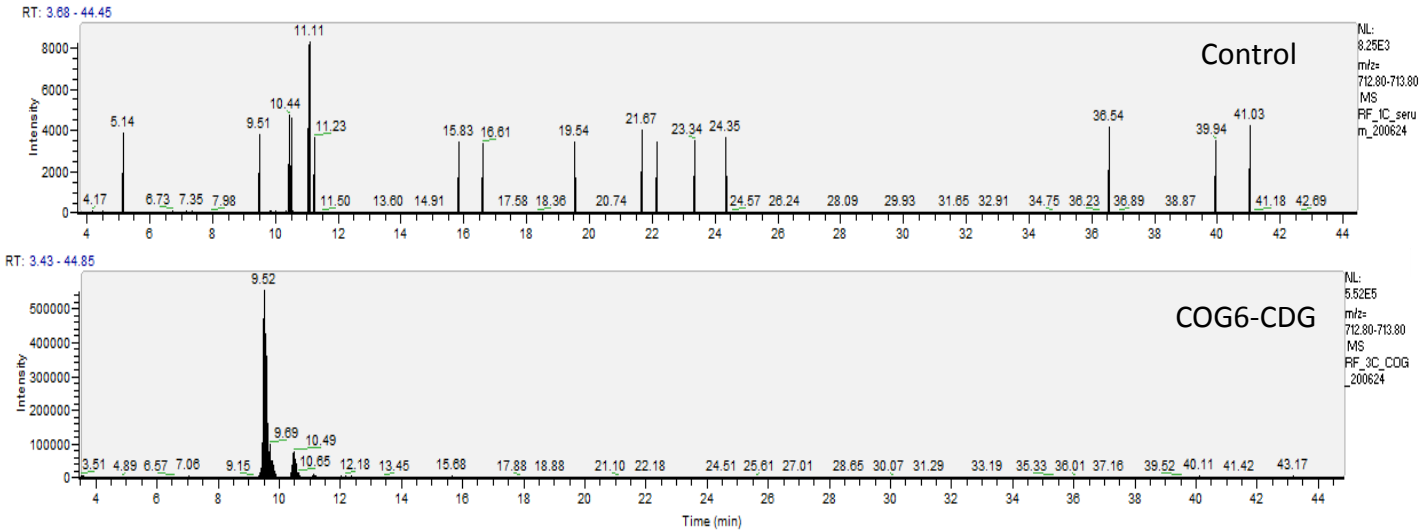

Hex3HexNAc4 (m/z 814.8)

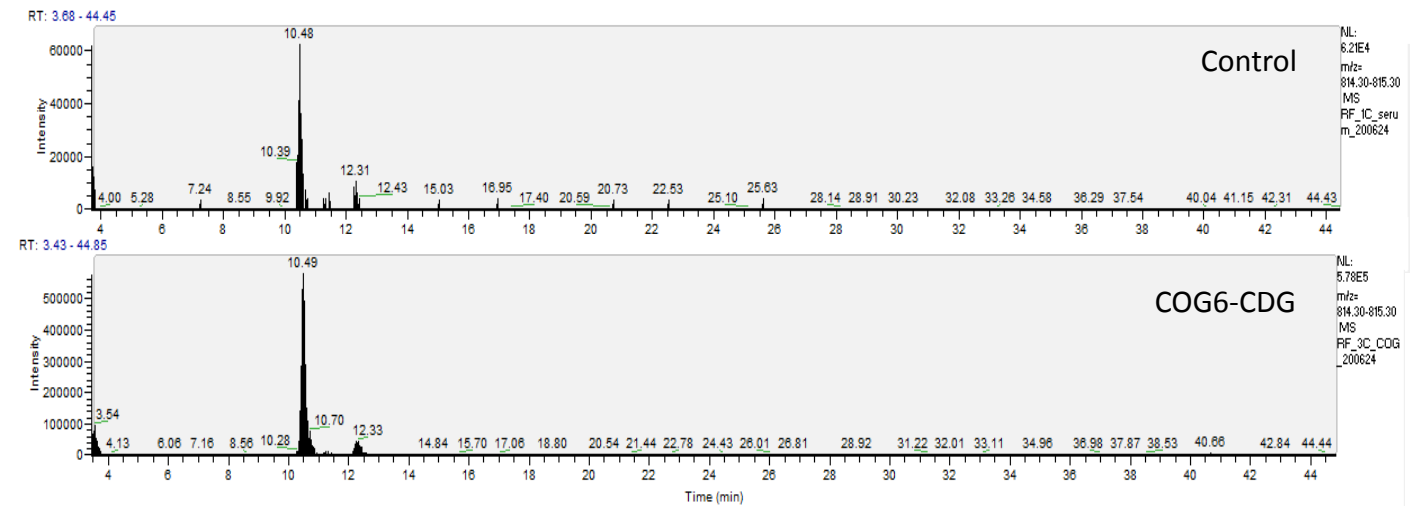

Supplement: Supporting Information 6 — Figure S6: Representative EICs of Hex3HexNAc3 and Hex3HexNAc4 N-glycans of both COG6-CDG patient and control. [file 7948771.f6.pdf]

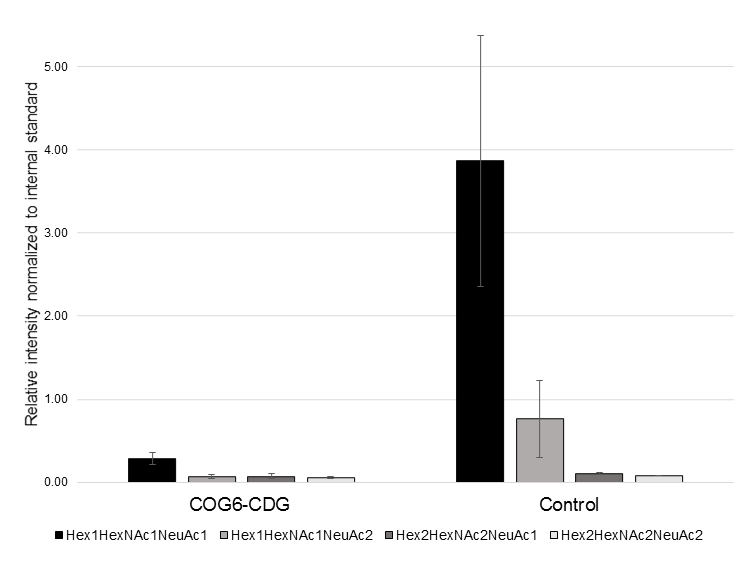

Supplement: Supporting Information 7 — Figure S7: Relative intensities of individual serum O-glycans of both COG6-CDG patient and control. Data are expressed as average ± SEM of two replicates. Hex, hexose; HexNAc, N-acetylhexosamine; NeuAc—sialic acid. [file 7948771.f7.tif]
